# Transcriptome profiling of resistant and susceptible Cavendish banana roots following inoculation with *Fusarium oxysporum* f. sp. *cubense* tropical race 4

**DOI:** 10.1186/1471-2164-13-374

**Published:** 2012-08-05

**Authors:** Chun-yu Li, Gui-ming Deng, Jing Yang, Altus Viljoen, Yan Jin, Rui-bin Kuang, Cun-wu Zuo, Zhi-cheng Lv, Qiao-song Yang, Ou Sheng, Yue-rong Wei, Chun-hua Hu, Tao Dong, Gan-jun Yi

**Affiliations:** 1 Institution of Fruit Tree Research, Guangdong Academy of Agricultural Sciences, Guangzhou, 510640, China; 2 Key Laboratory of South Subtropical Fruit Biology and Genetic Resource Utilization, Ministry of Agriculture, Guangzhou, 510640, China; 3 The college of Life Science, South China Agricultural University, Guangzhou, 510640, China; 4 Department of Plant Pathology, University of Stellenbosch, Private Bag X1, Matieland, 7602, South Africa

**Keywords:** Banana, *Fusarium oxysporum* f. sp. *cubense*, RNA-seq

## Abstract

**Background:**

Fusarium wilt, caused by the fungal pathogen *Fusarium oxysporum* f. sp. *cubense* tropical race 4 (Foc TR4), is considered the most lethal disease of Cavendish bananas in the world. The disease can be managed in the field by planting resistant Cavendish plants generated by somaclonal variation. However, little information is available on the genetic basis of plant resistance to Foc TR4. To a better understand the defense response of resistant banana plants to the Fusarium wilt pathogen, the transcriptome profiles in roots of resistant and susceptible Cavendish banana challenged with Foc TR4 were compared.

**Results:**

RNA-seq analysis generated more than 103 million 90-bp clean pair end (PE) reads, which were assembled into 88,161 unigenes (mean size = 554 bp). Based on sequence similarity searches, 61,706 (69.99%) genes were identified, among which 21,273 and 50,410 unigenes were assigned to gene ontology (GO) categories and clusters of orthologous groups (COG), respectively. Searches in the Kyoto Encyclopedia of Genes and Genomes Pathway database (KEGG) mapped 33,243 (37.71%) unigenes to 119 KEGG pathways. A total of 5,008 genes were assigned to plant-pathogen interactions, including disease defense and signal transduction. Digital gene expression (DGE) analysis revealed large differences in the transcriptome profiles of the Foc TR4-resistant somaclonal variant and its susceptible wild-type. Expression patterns of genes involved in pathogen-associated molecular pattern (PAMP) recognition, activation of effector-triggered immunity (ETI), ion influx, and biosynthesis of hormones as well as pathogenesis-related (PR) genes, transcription factors, signaling/regulatory genes, cell wall modification genes and genes with other functions were analyzed and compared. The results indicated that basal defense mechanisms are involved in the recognition of PAMPs, and that high levels of defense-related transcripts may contribute to Foc TR4 resistance in banana.

**Conclusions:**

This study generated a substantial amount of banana transcript sequences and compared the defense responses against Foc TR4 between resistant and susceptible Cavendish bananas. The results contribute to the identification of candidate genes related to plant resistance in a non-model organism, banana, and help to improve the current understanding of host-pathogen interactions.

## Background

Banana (including plantain) is the world’s principal fruit crop, with a production of over 100 million tons annually ( http://faostat.fao.org). However, the crop is threatened by Fusarium wilt, a fungal disease considered to be one of the most destructive in agricultural history [1,2]. Fusarium wilt, also known as Panama disease, is caused by the soil borne fungus, *Fusarium oxysporum* f. sp. *cubense* (Foc) [3]. The pathogen infects banana roots, colonizes and occludes the xylem vessels, and causes a reddish-brown discoloration of the rhizome and pseudostem. Leaves of infected banana plants eventually become bright yellow, before they wilt and collapse around the pseudostem [3]. Diseased banana plants often die before they produce bunches, thereby reducing yields in affected fields. Once the soil is infested with Foc, susceptible cultivars cannot be successfully replanted for up to 30 years [3].

Fusarium wilt has destroyed many thousands of hectares of bananas in tropical and subtropical countries [2,3]. Since the 1990s a damaging new variant of Foc, referred to as Foc tropical race 4 (TR4), has affected Cavendish bananas in the tropics of Asia and is considered a major threat to banana production worldwide [2]. Various control approaches have been used to combat or manage Fusarium wilt of banana. Among them, genetic resistance is regarded as the most effective and sustainable management option [4].

Understanding the complexity of disease resistance will contribute to the development of bananas that are resistant to Fusarium wilt. The genome sequence of banana is, however, largely unknown, as by April 24th, 2012 only about 118,277 banana EST sequences were released by the Global *Musa* Genomics Consortium and deposited in the NCBI databases. The publicly available DNA data are also not sufficient to explain the molecular mechanisms underlying resistance to Foc in banana. Therefore, extensive transcriptomic data are needed to discover genes related to Foc resistance. Such data could also serve as a good source for constructing high density microarrays for further characterization of gene expression profiles during banana/Foc interaction.

In this study, the transcriptome profiles of resistant and susceptible Cavendish banana roots infected with Foc TR4 were compared. Resistance was introduced into the Cavendish plants through the process of somaclonal variation [4]. More than 8 billion bases of high-quality DNA sequences were generated using Illumina technology, demonstrating the suitability of short-read sequencing for *de novo* assembly and annotation of genes expressed in non-model species without prior genome information. In the process, 169,950 non-redundant unigenes were identified, including hundreds of resistance-related, signaling, and metabolism genes. Furthermore, gene expression profiles of banana roots during different pathogen infection stages were compared using a digital gene expression **(**DGE) system.

## Results

### Determining the time-points for harvesting the samples

**Figure 1:**
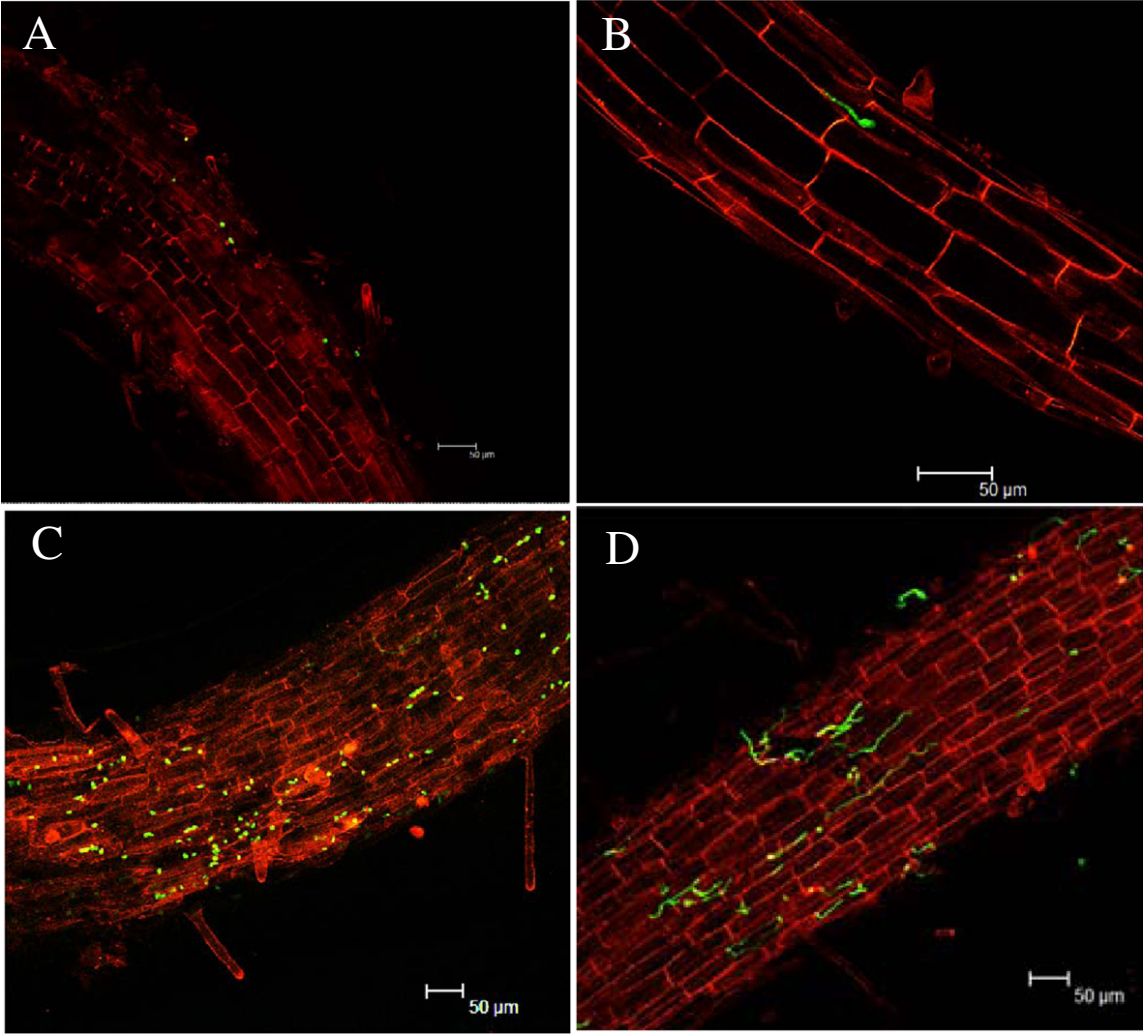
**Comparison of the colonization of *Fusarium oxysporum *f. sp. *cubense *tropical race 4 on the lateral roots of banana cv ‘Brazilian’ (susceptible wild-type) and cv ‘Nongke No 1’ (resistant mutant).** Few chlamydospores attached and germinated on the roots of cv ‘Nongke No 1’ at 48 hai (**A**) and 96 hai (**B**), respectively. Significant attachment and germination of spores of chlamydospores on the surface of the lateral roots of cv ‘Brazilian’ at 48 hai (**C**) and 96 hai (**D**) respectively; A-D. Scale bar = 50 μm.

The differences in disease progression between a resistant Cavendish banana mutant ‘Brazilian’ and its susceptible wild-type ‘Nongke No 1’ were monitored after infection with a GFP-tagged Foc TR4 isolate. Numerous spores were attached to the roots of ‘Brazilian’ 48 h after infection (hai), and most of these germinated and developed into hyphae 96 hai (Figure 1C and D). On ‘Nongke No 1’ roots, however, only a few fungal spores were found, and only a small number of these spores germinated at 96 hai (Figure 1A and B). Therefore, two time-points were selected to investigate the genetic basis underlying the differential responses of the two cultivars to infection, namely 48 and 96 hai.

### Comparative transcriptome analysis of ‘Brazilian’ and ‘Nongke No 1’

#### Illumina sequencing

**Figure 2:**
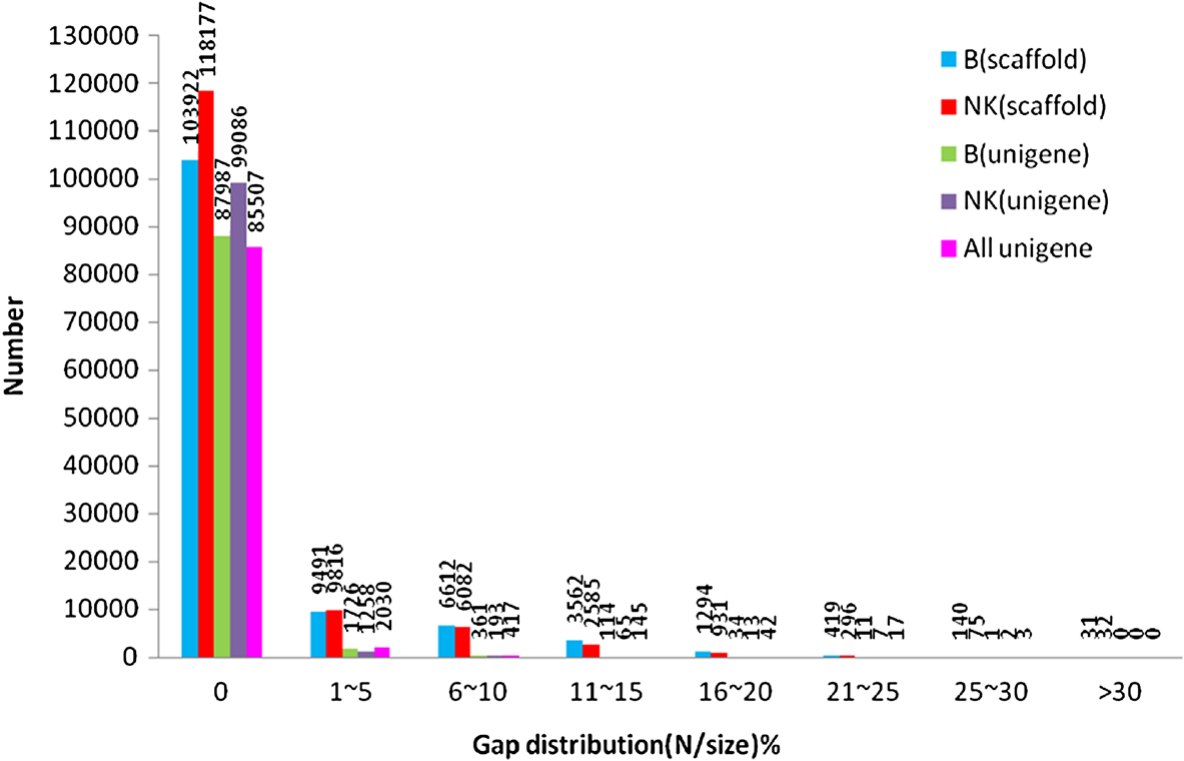
**Gap distribution among the scaffolds and unigenes found in ‘Brazilian’ (susceptible wild-type) and cv ‘Nongke No 1’ (resistant mutant) following inoculation with *Fusarium oxysporum *f. sp. *cubense *tropical race 4.**

Approximately 51.4 million and 51.9 million 90-bp clean paired end (PE) reads (Accession No. SRA049253.1) were generated for ‘Brazilan’ and ‘Nongke No 1’, respectively (Table 1). After the reads were assembled into contigs, scaffolds and clusters successively, they were analyzed for unigenes, of which more than 90,000 were present in ‘Brazilan’ and more than 100,000 in ‘Nongke No 1’ (Table 2). After the unigenes were merged for DGE analysis, 88,161 non-redundant unigenes that partially overlapped were removed (Accession No for B. JV310321-378292; Accession No for NK. JV378293- 451650). During *de novo* assembly, small gaps within the scaffolds containing the least number of unknown nucleotides (Ns) were filled in, permitting more than half of the gaps to be filled. The gap distribution for unigenes is shown in Figure 2.

**Table 1:** **Output statistics of the transcriptome of ‘Brazilian’ (susceptible wild-type) and cv ‘Nongke No 1’ (resistant mutant) inoculated with *Fusarium oxysporum *f. sp.*cubense *tropical race 4**

**Samples**	**Total reads**	**Total nucleotides**	**Q20 percentage**	**N percentage**	GC percentage*
B	51,447,154	4,630,243,860	92.82%	0.01%	52.86%
NK	51,943,226	4,674,890,340	93.12%	0.01%	52.03%

* Total Nucleotides = Total Reads1 x Read1 size + Total Reads2 x Read2 size, Q20 means base quality more than 20, N represents unknown nucleotides.

**Table 2:** **Output statistics of the Contig, Scaffolds and Unigenes of ‘Brazilian’ and ‘Nongke No 1’ bananas after inoculation with *Fusarium oxysporum *f. sp. *cubense *tropical race 4 **

	**Cotigs**				**Scaffolds**			**Unigenes**		
**Items**	**Numbers**	**Size distribution (bp)**	**Mean size (bp)**	**>100 bp(%)**	**Numbers**	**Mean size (bp)**	**>1000 bp(%)**	**Numbers**	**Size distribution (bp)**	**Mean size (bp)**
B	248,719	60 to 4,343	201	62.96	125,471	343	6.66	90,234	200 to 7503	425
NK	255,769	60 to 4,741	213	65.15	137,993	338	6.40	100,624	200 to 7503	414

#### Annotation of non-redundant unigenes

**Figure 3:**
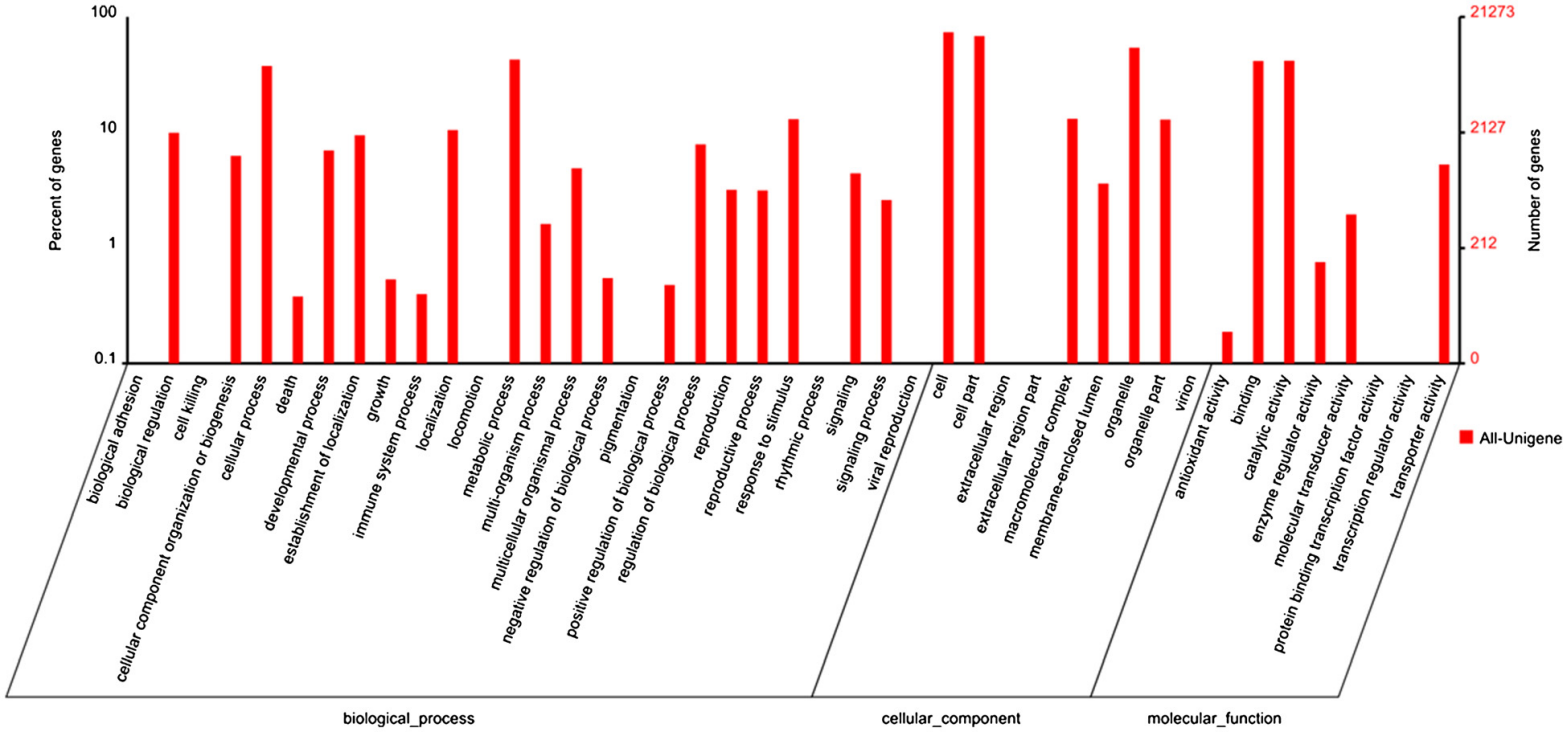
**GO annotations of unigenes found in ‘Brazilian’ (susceptible wild-type) and cv ‘Nongke No 1’ (resistant mutant) following inoculation with *Fusarium oxysporum *f. sp. *cubensev *tropical race 4.** The best hits were aligned to the GO database, and 45,723 transcripts were assigned to at least one GO term. Most consensus sequences were grouped into three major functional categories: biological process, cellular component and molecular function.

Among the 88,161 unigenes, 60,669 (68.82%) proved to be similar to known protein sequences from *Arabidopsis thaliana*, rice, maize, poplar, and *Pharbitis nil*. Annotation of the 60,669 sequences using Gene Ontology (GO) and Clusters of Orthologous Group (COG) databases yielded good results for approximately 45,722 unigenes and 50,410 putative proteins, respectively (Figure 3; Table 3).

**Table 3:** **COG annotations of putative proteins in Cavendish bananas following inoculation with *Fusarium oxysporum *f. sp.* cubense *tropical race 4.**

**COG function classification**	**Number of unigenes**	**Percentage**
General function prediction only	6577	13.05%
Translation, ribosomal structure, and biogenesis	5585	11.08%
Transcription	4649	9.22%
Posttranslational modification, protein turnover, chaperones	4266	8.46%
Replication, recombination and repair	3278	6.5%
Signal transduction mechanisms	3196	6.34%
Carbohydrate transport and metabolism	2913	5.78%
Function unknown	2825	5.60%
Cell cycle control, cell division, chromosome partitioning	2522	5.00%
Cell wall/membrane/envelope biogenesis	2359	4.68%
Amino acid transport and metabolism	1858	3.69%
Energy production and conversion	1598	3.17%
Intracellular trafficking, secretion, and vesicular transport	1434	2.84%
Inorganic ion transport and metabolism	1271	2.52%
Lipid transport and metabolism	1252	2.48%
Secondary metabolites biosynthesis, transport and catabolism	1167	2.32%
Cytoskeleton	879	1.74%
Coenzyme transport and metabolism	718	1.42%
Cell motility	434	0.86%
Nucleotide transport and metabolism	427	0.85%
Chromatin structure and dynamics	426	0.085%
Defense mechanisms	404	0.80%
RNA processing and modification	348	0.69%
Nuclear structure	13	0.03%
Extracellular structures	11	0.02%
Total	50,410	100%

#### Annotation of defense genes and pathways

**Figure 4:**
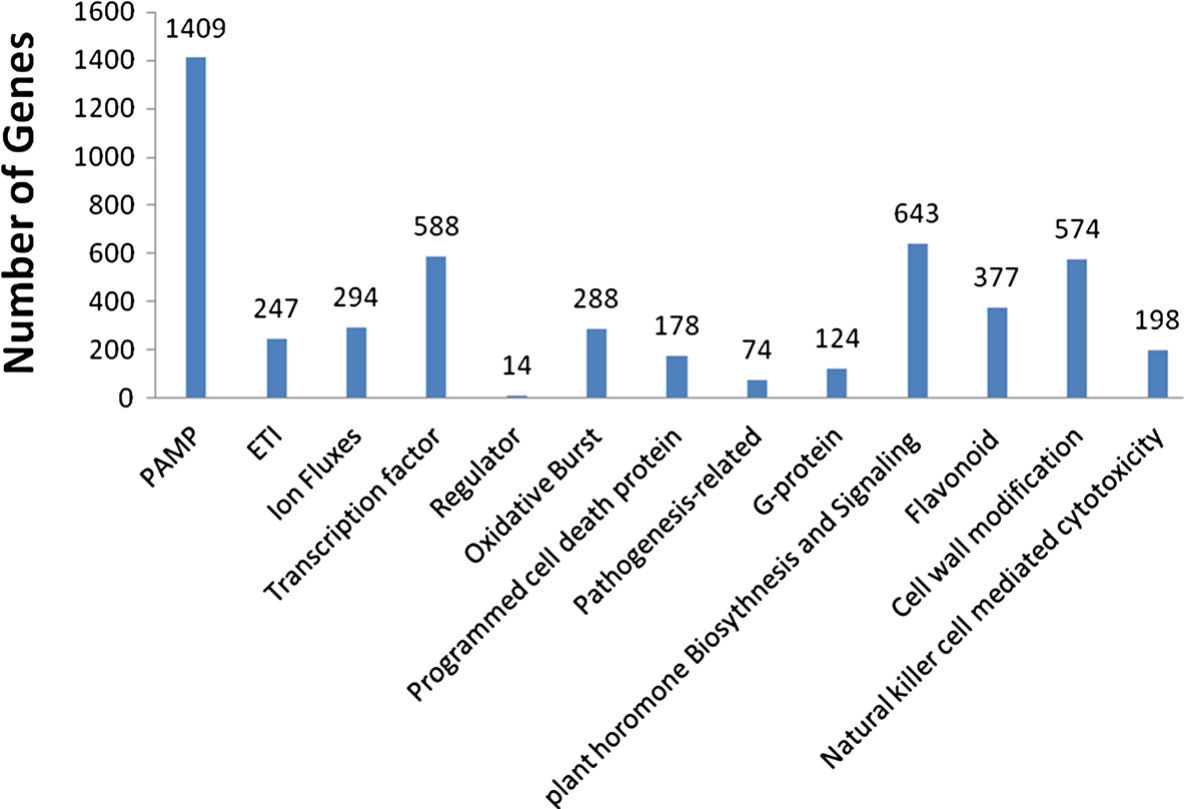
**Pathway assignment of defence-related genes in Cavendish banana following challenge with *Fusarium oxysporum *f. sp. *cubense *tropical race 4, based on KEGG p-values <0.0001; false discovery rate (FDR) ≤0.001; estimated absolute |log**_**2**_**Ratio| ≥ 1.**

To gain a deeper insight into the molecular biology of the defense system in banana, defense-related genes were analyzed. Approximately 5,008 unigenes were found to be homologous to known defense-related genes in other plants. KEGG analysis revealed that these unigenes were significantly enriched in various known resistance-relevant metabolic or signaling pathways (Figure 4; Additional file 1: Table S1), which suggests a considerable conservation of resistance-relevant genes and pathways between banana and other plants. These genes and pathways are required for both local and systemic acquired resistance. Thus, the involvement of these unigenes in metabolic pathways provides a basis for the further identification of biological functions of candidate genes in the banana responses.

#### Digital gene expression profile analysis after Foc inoculation

Illumina DGE of B, B1 and B2 (from ‘Brazilian’), and NK, NK1 and NK2 (from ‘Nongke No 1’) parallel sequencing resulted in 12.40, 12.09, 12.55, 12.50, 11.69 and 11.94 million high quality non-redundant tags (Accession No. SRA049253.1). Gene annotation by tag mapping analysis showed that 62.72%, 57.16%, 44.23%, 66.04%, 58.58% and 42.83% of all distinct tags in the six groups could be mapped to the reference database provided by the 88,161 non-redundant unigenes from the RNA sequence based transcriptome analysis.

**Figure 5:**
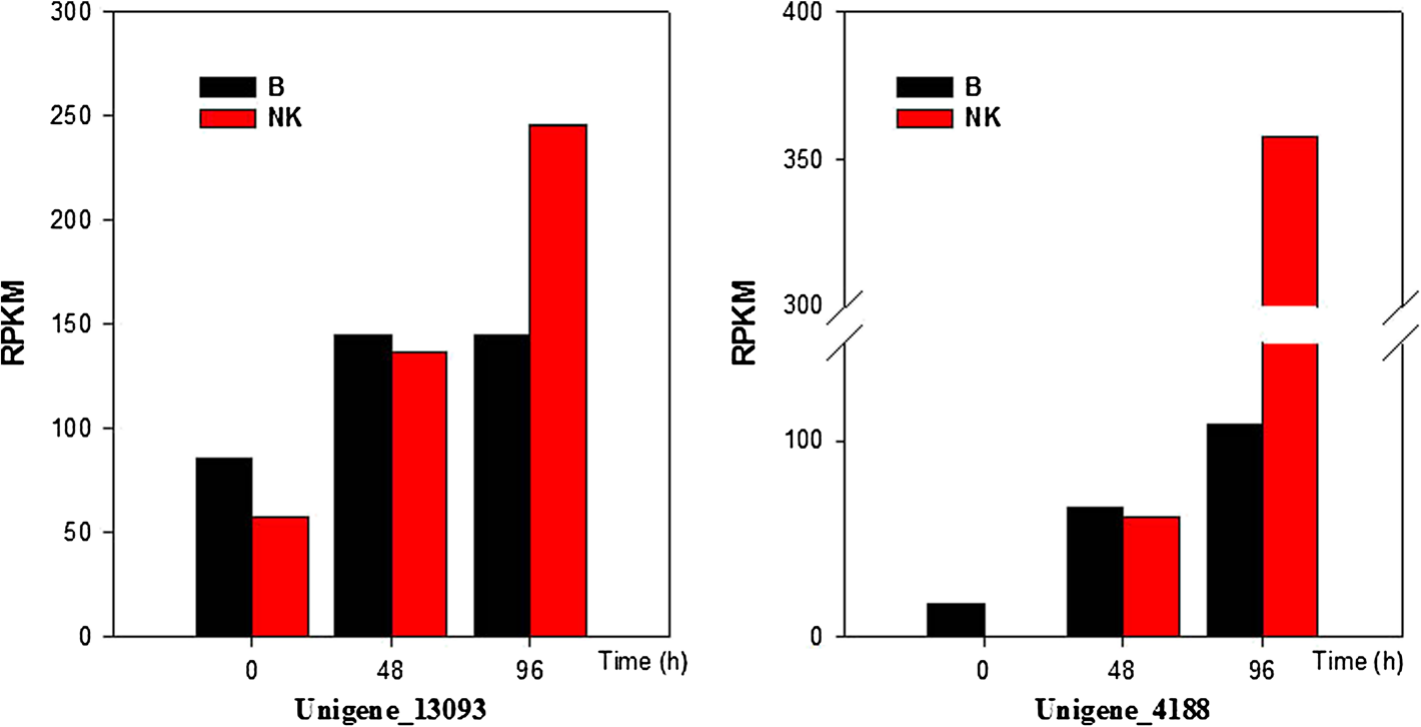
**Transcriptomic comparison of the unigenes CEBiP (left) and the Elicitor-responsive protein (right) between ‘Brazilian’ (susceptible wild-type) and cv ‘Nongke No 1’ (resistant mutant) following inoculation with *Fusarium oxysporum *f. sp. *cubense *tropical race 4.**

**Figure 6:**
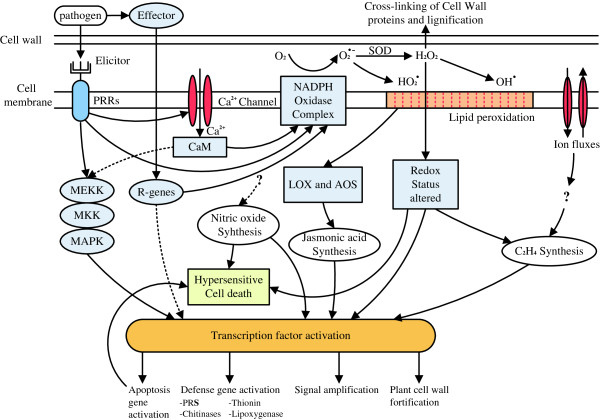
**Schematic representation of the response of the resistant Cavendish banana mutant ‘Nongke No 1’ against *Fusarium oxysporum *f. sp. *cubense *tropical race 4 (Foc TR4), adapted from D. Hofius et al. (2007).** The banana plant uses two strategies to respond to Foc TR4 attacks: PTI and ETI. The up-regulated PRRs include CEBIP and FLS2-like protein, and MAPK 5 and 12. Certain MEKK 2-like genes were also up-regulated, while MAPK 2, 6 and 10, MEK 4/5 and some MEKK 2-like genes were down-regulated. The up-regulated transcription factors include WRKY 6, 26, 31, 33, 65 and 72, and certain ERF-genes, while WRKY 56 and 75, BHLH 30, 25, 35 and 18, BIM 1 and 2, and HBP 1a were negative regulators. Except for the Ca^2+^ channel, ion fluxes also include potassium channel, Cl-channel (clc), and H^+^-transporting ATPases. Dotted lines and question marks represent uncertain and unknown pathways, respectively.

To analyze the global transcriptional changes in banana infected with Foc TR4, the method described by Audic et al. [5] was applied to identify differentially expressed genes from the normalized DGE data by pairwise comparisons between the wild-type (‘Brazilian’) and mutant banana (‘Nongke No 1’) at different time points after infection. To characterize the functional consequences of gene expression changes associated with infection with Foc in the resistant mutant, pathway analysis of differentially expressed genes was performed, based on the KEGG database using the two-side Fisher’s exact test. Nine metabolic pathways that are related to immunity were selected for further analysis. The selected pathways included Perception of PAMPs by Pattern Recognition Receptors (PRRs), Effector-triggered immunity (ETI), Ion fluxes, Transcription factors (TFs), Oxidative burst, Pathogenesis-related proteins (PRs), Programmed cell death (PCD), Plant hormones and Cell wall modification (Figure 4). Fifty-two types of differentially expressed genes with known or implicated functions were identified (Additional file 2: Table S2). All differentially expressed unigenes are involved in the recognition of PAMPs, and the high accumulated levels of defense-related transcripts may contribute to Foc TR4 resistance in banana (Additional file 2: Table S2; Additional file 3: Table S3). Transcriptomic comparison of two differentially expressed unigenes between’Brazilian’ and ‘Nongke No 1’ is shown in Figure 5. Based on the above results, a schematic illustration of plant defense in Cavendish banana against Foc TR4 was constructed (Figure 6). 

## Discussion

In this study, the plant defense response in banana following infection by the Fusarium wilt pathogen, Foc TR4, was investigated. The expression patterns of pathogen triggered immunity (PTI)- and effector triggered immunity (ETI)-related genes in response to Foc TR4 infection in the susceptible (wild-type) and resistant (its mutant) plant were compared using RNA-Seq. PTI constitutes the first layer of the plant defense response that restricts a pathogen from proliferating [6]. Most of the genes related to PTI in banana exhibited different expression patterns and levels in the resistant mutant (‘Nongke No 1’) relative to its wild-type (‘Brazilian’). For instance, the chitin elicitor-binding protein (CEBiP) and the chitin elicitor receptor kinase (CERK1); both important components of the plant signaling pathway that recognizes chitin oligosaccharides, a representative general elicitor inducing defense responses in a wide range of plant cells of both monocots and dicots [7], were up-regulated in ‘Nongke No 1’ compared to ‘Brazilian’. Knockout mutants of either of these genes should be performed to elucidate their role in the defense of banana against Foc in future, as it was previously demonstrated that their disruption resulted in the partial impairment of the ability of rice to respond to the chitin elicitor of an incompatible fungus, including MAPK activation, ROS generation, and disease resistance, [7].

Brassinosteroid Insensitive 1-Associated Kinase 1 (BAK1) acts in PTI via its interaction with Flagellin Sensing 2 (FLS2). Most known PRRs require BAK1 for function [8]; BAK1 is known as Somatic Embryogenesis Receptor-Like Kinase 3 (SERK3) [9] and does not have a direct role in elicitor perception, but FLS2 rapidly forms a complex with it after elicitation. This interaction results in phosphorylation of both proteins [10]. In *Nicotiana benthamiana*, the BAK1/SERK homolog has a direct role in elicitor perception of bacterial cold shock protein, flagellin, and elicitin, but not chitin [8]. Although we do not know whether similar elicitors exist in Foc TR4, in the expression profile, both FLS2 and BAK1 had the same expression pattern in both cultivars. Both of them were up-regulated upon inoculation in the mutant, and their expression levels were several times those of the wild-type, which indicated that the FLS2–BAK1 complex might interfere with the activation of banana innate immunity.

A common prerequisite in plant innate immunity is elicitor-stimulated activation of cyclic nucleotide gated channels (CNGCs)-Ca^2+^ influx, which initiates all subsequent defense reactions [11]. CNGC 2, CNGC 4, CNGC 11 and CNGC 12 mediate this influx after elicitor perception in *Arabidopsis*[12]. However, in *Musa*, we found that CNGC 1, CNGC 5 and CNGC 6 changed after PAMP perception, which suggested that there is a fundamental difference in the Ca^2+^ influx mechanism between banana and *Arabidopsis*.

Compared to the significant increase in the expression of PTI genes in the resistant ‘Nongke No 1’ banana, the expressions of most R genes, such as cc-nbs-lrr resistance protein, NBS-type resistance protein, were very low, except for the RIN4/RPM1 complex. In *A. thaliana*, RPM1 conferred resistance to *Pseudomonas syringae* expressing either *avrRpm1* or *avrB*[13]. AvrB and AvrRpm1 cause hyperphosphorylation of the RPM1 interacting protein 4 (RIN4) [14], and these modifications are perceived by RPM1, which subsequently triggers disease resistance [15]. The different expression of the RIN4/RPM1 complex in the resistant Cavendish banana compared to the susceptible wild type may be one way of explaining the resistance in the mutant plant. . This result is consistent with findings in the knockout mutant of *A. thaliana* (REF). Although RPS2 was expressed at a low level in banana, elimination of RIN4 or inhibition of the expression of RIN4 by unknown effectors from Foc TR4 will activate the RPS2 pathway. The effectors secreted by Foc TR4 remain unknown, and the presence of a similar effector to AvrRpt2 requires further investigation.

PAMPs and ETI are known to induce rapid production of ROS (Reactive oxygen species) in an oxidative burst after treatment with a pathogen, which is largely derived from the activity of membrane-localized NADPH oxidases [15]. NADPH oxidases (or Respiratory burst oxidase) were activated and up-regulated in the resistant banana in response to Foc TR4 infection, which is in agreement with previous observations in wheat, cotton, and cucumber after infection by the Fusarium wilt fungal pathogen [16]. We also investigated the expression of ROS-scavenging systems, such as Catalase, Ascorbate peroxidase and etc., and found that most of them had a higher expression in the susceptible wild-type. This suggested that there was a higher level of ROS in the mutant, which inhibited the colonization of the pathogen on the root.

Both BAGs and AIF had higher expression levels in the susceptible cultivar than in the resistant mutant, which indicated that PCD was increased by Foc TR4 attack in the wild-type, which conflicts with the low concentration of ROS. Delledonne [17] reported that Nitrogen Monoxide (NO) and ROS together, but not individually, are required to induce HR-mediated cell death, and that the balance between NO and H_2_O_2_ needs to be further investigated [18].

The salicylic acid (SA), jasmonic acid (JA) and ethylene (ET) hormone pathways are important regulators of defense-gene expression [19]. We first analyzed the SA signaling-related genes, and did not find significant differences between the two cultivars, which indicated that it did not play a role in the resistance response and was in agreement with the suggestion that SA is not involved in resistance to necrotrophic pathogens, such as Foc TR4. The core JA-signaling component jasmonate ZIM-motif (JAZ) proteins TIFY10B-like gene and a jasmonate inducible protein, extracellular superoxide dismutase [Cu-Zn]^2+^, were induced in the mutant at all time points, while it was expressed in the wild-type only in trace amounts. Additionally, the expression levels of Lipoxygenase (LOX)-like and Allene oxide synthase (AOS)-like unigenes indicated that endogenous levels of JA in the resistant mutant were much higher than in the wild-type. For the ethylene signaling genes, such as transcription factors Ethylene Insensitive 3 (EIN3) and Ethylene Insensitive 3-like 1 (EIL1), transcription levels in the mutant plant were much higher than those in the wild-type. This suggests that resistance to the necrotrophic pathogen Foc TR4 is mediated by the JA and ET signaling pathways, and not the SA pathway.

The plant cell wall not only serves as a physical barrier, but also as a defense barrier against pathogen penetration. Expressions of 3-Deoxy-d-arabino-heptulosonate-7-phosphate synthase (DAHPS), 4-coumarate: CoA ligase (4CL), polyphenol oxidase (PPO), glutathione S-transferase (GST), UDP-glucuronic acid decarboxylase and cellulose synthase, which act at different steps of the shikimate-phenylpropanoid-lignin and cellulose biosynthesis pathways, were up-regulated in the compatible interaction after infection of Foc TR4. Their expression, however, was not affected in the incompatible interaction. This result is not consistent with previous reports [20], which proved that the above pathways were induced in incompatible infection, but did not change or was repressed in the compatible interaction. In wheat and watermelon, resistance to necrotrophic fungus is executed after penetration, and the density of the intercellular hyphae and the number of haustoria were greatly reduced in an incompatible compared with the compatible interaction (REF). However, in banana, resistance to Foc TR4 appeared to occur before colonization, as was demonstrated in the current study where the amount of spores attached to roots of the resistant mutant was substantially reduced. In the susceptible ‘Brazilian’ banana, however, a large amount of Foc TR4 spores colonized and germinated on the roots.

## Conclusions

In this study, we characterized the root transcriptome of banana and provided a comparative DGE analysis of the compatible and incompatible interaction between banana and Foc TR4. These findings provide a substantial contribution to existing sequence resources for banana, and a strong basis for future genomic research. The differentially expressed genes and putative signaling pathways generated in the present study revealed that the defense system of banana may be more complex than previously believed. The findings of this study will hopefully accelerate research on resistance in banana to Foc TR4 and contribute to a better understanding of the banana defense response to plant pathogens. Many defense-related genes and pathways in banana differ from those in model plants such as rice and *Arabidopsis*, suggesting that the mechanisms underlying host defense in plants may be variable. To the best of our knowledge, this is the first use of Illumina sequencing technology for banana root transcriptome *de novo* sequencing and assembly without a reference genome. Among the generated sequences, 2,691 unigenes were specifically expressed in the incompatible interaction. These genes could play an important role in the interaction of banana and Foc TR4, and their spatial and temporal expressions require further study.

## Methods

### Plant material and inoculation

Micropropagated Cavendish banana plantlets of the Foc TR4-susceptible variety ‘Brazilian’ and its resistant mutant ‘Nongke No 1’ were used. Plantlets with four or five leaves and approximately 30 cm in height were transplanted into sterile medium that consisted of three parts vermiculite, one part peat and 0.5 parts coconut coir. All plantlets were kept in a greenhouse at 25-32°C with a 16-h light/8-h dark photoperiod.

For inoculation, a GFP-tagged Foc TR4 [21] and its wild-type isolate (CGMCCC 3.12196, VCG 01213) with the similar growth characteristics and similar virulence were used. The GFP-tagged transformant was used to observe the infection process and determine the time-points for RNA extraction, while the wild-type isolate was used to inoculate roots for RNA extraction. The method used for plant inoculation was described before [21], except that a final inoculation concentration 5000 conidia/g soil was used. Plants were sampled at 48 and 96 hai. The samples were marked as B, B1, B2, NK, NK1, and NK2.

### Sample preparation, cDNA library construction, and Illumina sequencing

Total RNA was extracted from Cavendish banana roots using the QIAGEN RNeasy plant mini kit (QIAGEN, Valencia, CA), and treated with RNase-free DNAse I (Fermentas Life Sciences, Hanover, MD). RNA integrity was confirmed using the 2100 Bioanalyzer (Agilent Technologies) with a minimum RNA integrated number value of eight. Two mixtures of equal amounts of the three banana root RNA samples for transcriptome analysis was prepared for each cultivar respectively using Illumina’s kit, following the manufacturer’s recommendations. Briefly, mRNA was purified from 30 μg of total RNA using Sera-mag Magnetic Oligo (dT) Beads (Illumina) and fragmented into small pieces using divalent cations under elevated temperature. Double-stranded cDNA was then synthesized using the SuperScript double-stranded cDNA Synthesis kit (Invitrogen, Camarillo, CA) with random hexamer (N6) primers (Illumina). These cDNA fragments then went through an end repair process, phosphorylation, and ligation of adapters. Products were subsequently purified and amplified by PCR to create the final cDNA libraries. The insert size of the library was approximately 200 bp. The cDNA library was sequenced on the Illumina HiSeq™ 2000, and both ends of the inserts were sequenced. The 90-bp raw PE reads were generated by the Illumina Genome Analyzer II system.

### Data filtering and *de novo* assembly of the transcriptome

A stringent filtering process was carried out. The original image data were transferred into sequence data by base calling, which was defined as raw data or raw reads, and saved as fastq files. To obtain clean reads, dirty raw reads were removed, such as reads with adaptors, reads in which unknown bases were more than 10%, and low quality reads (the percentage of the low quality bases of quality value ≤ 5 was more than 50% in a read). *De novo* assembly was carried out using SOAPdenovo. SOAPdenovo first combined the high quality reads into contigs, and then connected them into scaffolds. Finally, paired-end reads were used for gap filling of the scaffolds to obtain sequences with the least number of Ns and that could not be extended on either end. These were defined as unigenes. The sequences were used for blast searching and annotation using BLASTx programs (e-value < 0.00001) against the NCBI non-redundant (nr) protein database [22]. Functional annotation by gene ontology terms [23] was analyzed by Blast2GO program (Conesa et al., 2005 and 2008). The COG and KEGG pathways annotation was performed using Blastall software against Cluster of Orthologous Groups database [24] and the Kyoto Encyclopedia of Genes and Genomes (KEGG) pathway database [25].

### Digital gene expression profiling

RNA was extracted from six banana samples; three from Brazilian (B, B1, B2), and three from Nongke No 1 (NK, NK1 and NK2). A tag library was then constructed using DGE. For Tag Profiling, the Nla III Sample Prep kit from Illumina was used according to the manufacturer’s instructions. In brief, mRNA was captured from 1 μg of total RNA using magnetic oligo (dT) beads. Double-strand cDNA was synthesized and bead-bound cDNA was subsequently digested with Nla III. Fragments other than the 3’ cDNA fragments attached to oligo (dT) beads were washed away and a GEX Nla III adapter was ligated to the free 5’ end of the digested bead-bound cDNA fragments. The GEX Nla III adapter contains a restriction site for Mme I which cuts 17–18 bp downstream from the Nla III site, thereby releasing 21–22 bp tags starting with the Nla III recognition sequence, CATG. A second adapter (GEX adapter 2) was ligated at the site of Mme I cleavage, and the adapter-ligated cDNA tags were enriched by linear PCR amplification. The resulting 85-bp fragments were purified from a 6% acrylamide gel. The cDNA was then digested, and the single-chain molecules were fixed onto the Illumina sequencing chip for sequencing.

For annotation, all tags were mapped to the reference sequences and no more than one nucleotide mismatch was allowed. All the tags mapped to reference sequences from multiple genes were filtered and the remaining tags were designated as unambiguous tags. For gene expression analysis, the number of expressed tags was calculated and then normalized to TPM (number of transcripts per million tags); and the differentially expressed tags were used for mapping and annotation.

### Statistical analysis

A statistical analysis of the frequency of each tag in the different cDNA libraries was performed to compare gene-expression in different stages. Statistical comparison was performed with custom written scripts using the method described by Audic et al. [5]. The false discovery rate (FDR) was used to determine the threshold of P value in multiple test and analysis. A threshold of FDR < 0.001 was used to judge the significance of gene expression difference. In this research, P ≤ 0.01, FDR ≤ 0.1, and the absolute value of log2Ratio ≥ 1 were used as threshold to assess the significance of gene expression difference.

## Competing interests

The authors declare that they have no competing interests.

## Authors’ contributions

CL and GY conceived and designed the study, and wrote the manuscript. GD and JY inoculated the banana plantlets, extracted RNA and participated in the bioinformatics analysis. YJ drew the picture of Figure 7. Altus Viljoen took part in writing the paper. RK, CZ, ZL, QY, OS, YW, CH, TD drafted the manuscript, designed the tables, reviewed the manuscript, and provided guidance. All authors read and approved the final manuscript.

## Supplementary Material

Additional file 1 **Table S1.**Immunity-related unigenes in the transcriptome of ‘Brazilian’ (susceptible wild-type) and cv ‘Nongke No 1’ (resistant mutant) bananas following inoculation with *Fusarium oxysporum* f. sp. *cubense* tropical race 4. Click here for file

Additional file 2 **Table S2.**List of metabolic pathways related to host defense in banana following inoculation with *Fusarium oxysporum* f. sp. *cubense* tropical race 4 [7,13,26-70]. Click here for file

Additional file 3 **Table S3.**Expression of unigenes in ‘Brazilian’ (susceptible wild-type) and cv ‘Nongke No 1’ (resistant mutant) bananas following inoculation with *Fusarium oxysporum* f. sp. *cubense* tropical race 4. Click here for file
