# Supplementary material for: Transcriptome profiling of resistant and susceptible Cavendish banana roots following inoculation with Fusarium oxysporum f. sp. cubense tropical race 4
Source: BMC Genomics. 2012 Aug 5;13:374. doi: 10.1186/1471-2164-13-374 (PMC3473311; doi:10.1186/1471-2164-13-374)
Supplement: Additional file 1 — Table S1.Immunity-related unigenes in the transcriptome of ‘Brazilian’ (susceptible wild-type) and cv ‘Nongke No 1’ (resistant mutant) bananas following inoculation with Fusarium oxysporum f. sp. cubense tropical race 4. [file 1471-2164-13-374-S1.doc]

Additional file 1, Table S1

|  | | | | | | | **Number** |
| --- | --- | --- | --- | --- | --- | --- | --- |
| **PAMP** | | | | | | | 1409 |
|  | 1.the Receptor-like Kinases | | | | | | 1162 |
|  |  | | | | 1.wall-associated receptor kinase | | 42 |
|  |  |  |  |  |  | WAK1 | 1 |
|  |  |  |  |  |  | WAK2 | 9 |
|  |  |  |  |  |  | WAK3 | 2 |
|  |  |  |  |  |  | WAK5 | 5 |
|  |  |  |  |  |  | WAKL5 | 2 |
|  |  |  |  |  |  | WAKL9 | 9 |
|  |  |  |  |  |  | WAKL10 | 2 |
|  |  |  |  |  |  | WAKL14 | 8 |
|  |  |  |  |  |  | WAKL20 | 2 |
|  |  |  |  |  |  | WAKL22 | 1 |
|  |  | | | | 2.Leu-rich repeat receptor kinase(LRR-RK) | | 128 |
|  |  |  |  |  |  | RPK2（LRR receptor-like serine/threonine-protein kinase RPK2） | 18 |
|  |  |  |  |  |  | FLS2（LRR receptor-like serine/threonine-protein kinase FLS2） | 9 |
|  |  |  |  |  |  | MRH1（Probable LRR receptor-like serine/threonine-protein kinase MRH1） | 11 |
|  |  |  |  |  |  | GSO1（LRR receptor-like serine/threonine-protein kinase GSO1） | 14 |
|  |  |  |  |  |  | GSO2（LRR receptor-like serine/threonine-protein kinase GSO2） | 5 |
|  |  |  |  |  |  | PBS1（Serine/threonine-protein kinase PBS1） | 36 |
|  |  |  |  |  |  | ERL2(LRR receptor-like serine/threonine-protein kinase ERL2) | 2 |
|  |  |  |  |  |  | EFR（LRR receptor-like serine/threonine-protein kinase EFR） | 9 |
|  |  |  |  |  |  | ERECTA（LRR receptor-like serine/threonine-protein kinase ERECTA） | 17 |
|  |  |  |  |  |  | FEI1（LRR receptor-like serine/threonine-protein kinase FEI 1） | 6 |
|  |  |  |  |  |  | RKF3（Probable LRR receptor-like serine/threonine-protein kinase RKF3） | 1 |
|  |  |  |  |  | 3. somatic embryogenesis receptor kinase(SERK) | | 34 |
|  |  |  |  |  |  | somatic embryogenesis receptor kinase 1 | 16 |
|  |  |  |  |  |  | somatic embryogenesis receptor kinase 4 | 2 |
|  |  |  |  |  | 4.chitin elicitor receptor kinase 1 | | 32 |
|  |  |  |  |  | 5.brassinosteroid insensitive 1-associated receptor kinase 1(BAK1) | | 5 |
|  |  |  |  |  | 6. brassinosteroid insensitive 1(BRI1) | | 5 |
|  |  |  |  |  | 7.Phytosulfokine receptor 1 | | 22 |
|  |  |  |  |  | 8.RLK(Receptor-like protein kinase) | | 28 |
|  |  |  |  |  | 9.CPK6(Calcium-dependent protein kinase 6) | | 6 |
|  |  |  |  |  | 10.Proline-reich extensin-like receptor kinase(PERK) | | 151 |
|  |  |  |  |  |  | PERK1 | 32 |
|  |  |  |  |  |  | PERK8 | 31 |
|  |  |  |  |  |  | PERK10 | 14 |
|  |  |  |  |  |  | PERK13 | 7 |
|  |  | | | |  | PERK14 | 10 |
|  |  |  |  |  |  | Others | 57 |
|  |  |  |  |  | 11.LysM receptor-like kinase | | 4 |
|  |  |  |  |  | 12.Cysteine-rich receptor-like protein kinase | | 50 |
|  |  |  |  |  | 13.interleukin-1 receptor-associated kinase(IRAK) | | 73 |
|  |  |  |  |  | 14.nodulation receptor kinase-like protein | | 8 |
|  |  |  |  |  | 15.Probable inactive receptor kinase | | 54 |
|  |  |  |  |  | 16.inactive leucine-rich repeat receptor-like protein kinase | | 3 |
|  |  |  |  |  | 17.leucine-rich repeat receptor-like protein kinase | | 144 |
|  |  |  |  |  | 18.LRR receptor-like serine/threonine-protein kinase | | 330 |
|  |  |  |  |  | 19.Lectin-domain containing receptor kinase | | 39 |
|  |  |  |  |  | 20.S-receptor kinase-like | | 4 |
|  | 2.Chitin elicitor-binding protein | | | | | | 2 |
|  | 3.Elicitor-responsive protein | | | | | | 8 |
|  | 4. Mitogen-activated protein kinase | | | | | | 133 |
|  |  | | | | Mitogen-activated protein kinase 1 | | 7 |
|  |  |  |  |  | Mitogen-activated protein kinase 2 | | 6 |
|  |  |  |  |  | Mitogen-activated protein kinase 4 | | 7 |
|  |  |  |  |  | Mitogen-activated protein kinase 5 | | 4 |
|  |  |  |  |  | Mitogen-activated protein kinase 6 | | 1 |
|  |  |  |  |  | Mitogen-activated protein kinase 7 | | 3 |
|  |  |  |  |  | Mitogen-activated protein kinase 9 | | 5 |
|  |  |  |  |  | Mitogen-activated protein kinase 10 | | 12 |
|  |  |  |  |  | Mitogen-activated protein kinase 12 | | 3 |
|  |  |  |  |  | Mitogen-activated protein kinase 18 | | 1 |
|  |  |  |  |  | NTF4 | | 1 |
|  |  |  |  |  | Mitogen-activated protein kinase 20 | | 3 |
|  |  |  |  |  | Mitogen-activated protein kinase homolog D5 | | 1 |
|  |  |  |  |  | Mitogen-activated protein kinase kinase kinase A | | 13 |
|  |  |  |  |  | Mitogen-activated protein kinase kinase kinase 1 | | 13 |
|  |  |  |  |  | Mitogen-activated protein kinase kinase kinase 2 | | 8 |
|  |  |  |  |  | Mitogen-activated protein kinase kinase kinase ANP1 | | 4 |
|  |  |  |  |  | mitogen-activated protein kinase kinase1 | | 5 |
|  |  |  |  |  | mitogen-activated protein kinase kinase 2 | | 6 |
|  |  |  |  |  | mitogen-activated protein kinase kinase 4 | | 8 |
|  |  |  |  |  | mitogen-activated protein kinase kinase 4/5 | | 15 |
|  |  |  |  |  | mitogen-activated protein kinase kinase 5 | | 5 |
|  |  |  |  |  | mitogen-activated protein kinase kinase 6 | | 1 |
|  |  |  |  |  | mitogen-activated protein kinase kinase 10-2 | | 1 |
| **ETI** | | | | | | | 247 |
|  | 1.resistance genes (R-genes) | | | | | | 25 |
|  |  | | | | 1. cc-nbs-lrr resistance protein | | 20 |
|  |  |  |  |  | 2. serine-threonine protein kinase | | 5 |
|  | 2. R-gene interacting proteins | | | | | | 68 |
|  |  | | | | Disease resistance protein RPM1 | | 12 |
|  |  |  |  |  | RPM1-interacting protein 4(RIN4) | | 20 |
|  |  |  |  |  | RSP2 | | 22 |
|  |  |  |  |  | NSP-interacting kinase 1 | | 1 |
|  |  |  |  |  | Pto-interacting protein 1 | | 10 |
|  |  |  |  |  | CBL-interacting protein kinase 10 | | 2 |
|  |  |  |  |  | Rop-interacting receptor-like cytoplasmic kinase 2 | | 1 |
|  |  |  |  |  | phytochrome-interacting factor 3 | | 51 |
|  | 3.others | | | | | | 154 |
|  |  | | | | (1).RPP13 | | 3 |
|  |  |  |  |  | (2).RGA2 | | 74 |
|  |  |  |  |  | (3).RGC2 | | 1 |
|  |  |  |  |  | (4).RGC5 | | 43 |
|  |  |  |  |  | (5).RSP5 | | 26 |
|  |  |  |  |  | (6).ADR1 | | 2 |
|  |  |  |  |  | (7).SH20 | | 1 |
|  |  |  |  |  | (8).Rps1-k-1 | | 1 |
|  |  |  |  |  | (9).RPH8A | | 1 |
|  |  |  |  |  | (10).RGH1-like | | 2 |
| **Ion Fluxes** | | | | | | | **294** |
|  | | 1. Calcium/calmodulin | | | | | 34 |
|  |  |  | | | CML11(Calmodulin-like protein ) | | 7 |
|  |  |  |  |  | CML22 | | 2 |
|  |  |  |  |  | CML23 | | 2 |
|  |  |  |  |  | CML36 | | 6 |
|  |  |  |  |  | CML37 | | 2 |
|  |  |  |  |  | CML38 | | 2 |
|  |  |  |  |  | CML41 | | 4 |
|  |  |  |  |  | CML45 | | 4 |
|  |  |  |  |  | CML49 | | 5 |
|  |  | 2.Calcium-dependent protein kinase | | | | | 103 |
|  |  |  | | | CPK1(Calcium-dependent protein kinase 1) | | 8 |
|  |  |  |  |  | CPK3(Calcium-dependent protein kinase 3) | | 8 |
|  |  |  |  |  | CPK4(Calcium-dependent protein kinase 4) | | 10 |
|  |  |  |  |  | CPK7(Calcium-dependent protein kinase 7) | | 2 |
|  |  |  |  |  | CPK8(Calcium-dependent protein kinase 8) | | 6 |
|  |  |  |  |  | CPK13(Calcium-dependent protein kinase 13) | | 9 |
|  |  |  |  |  | CPK16(Calcium-dependent protein kinase 16) | | 3 |
|  |  |  |  |  | CPK19(Calcium-dependent protein kinase 19) | | 2 |
|  |  |  |  |  | CPK29(Calcium-dependent protein kinase 29) | | 4 |
|  |  |  |  |  | CPK32(Calcium-dependent protein kinase 32) | | 6 |
|  |  |  |  |  | CPK34(Calcium-dependent protein kinase 34) | | 4 |
|  |  | 3.AK1 | | | | | 5 |
|  |  | 4.calcium-binding EF hand family protein | | | | | 21 |
|  |  | 5.SCaMC-1(Calcium-binding mitochondrial carrier protein SCaMC-1) | | | | | 8 |
|  |  | 6.(Calcium and calcium/calmodulin-dependent serine/threonine-protein kinase) | | | | | 1 |
|  |  | 7.MCK2(calcium/calmodulin dependent protein kinase MCK2) | | | | | 1 |
|  |  | 8.ZmCPK11(calcium-dependent protein kinase ZmCPK11) | | | | | 1 |
|  |  | 9.SK5（Calcium-dependent protein kinase SK5） | | | | | 10 |
|  |  | 10.CNGC | | | | | 39 |
|  |  |  | | CNGC1 | | | 4 |
|  |  |  |  | CNGC2 | | | 8 |
|  |  |  |  | CNGC4 | | | 8 |
|  |  |  |  | CNGC5 | | | 4 |
|  |  |  |  | CNGC6 | | | 4 |
|  |  |  |  | CNGC14 | | | 3 |
|  |  |  |  | CNGC16 | | | 2 |
|  |  |  |  | CNGC17 | | | 4 |
|  |  |  |  | CNGC20 | | | 2 |
|  |  | 11.voltage-gated potassium channel | | | | | 12 |
|  |  | 12.NKT5 | | | | | 1 |
|  |  | 13.AKT1 | | | | | 7 |
|  |  | 14.AKT2 | | | | | 5 |
|  |  | 15.SKOR | | | | | 6 |
|  |  | 16.Cl-channel (clc-7) | | | | | 3 |
|  |  | 17.NaCl-inducible protein | | | | | 2 |
|  |  | 18.F-type H+-transporting ATPase subunit delta | | | | | 23 |
|  |  | 19.Vacuolar H+-ATPase V1 sector, subunit C | | | | | 12 |
| **Transcription factor** | | | | | | | **588** |
|  | 1.WRKY | | | | | | 241 |
|  | 2.BHLH | | | | | | 196 |
|  | 3.Ethylene-responsive transcription factor 1 | | | | | | 40 |
|  | 4.FIT(Transcription factor FER-like iron deficiency-induced transcription factor) | | | | | | 1 |
|  | 5.TT8(Transcription factor TT8) | | | | | | 8 |
|  | 6.UNE12 | | | | | | 4 |
|  | 7.RAP1 | | | | | | 14 |
|  | 8.HBP-1a | | | | | | 14 |
|  | 9.MYC7E | | | | | | 3 |
|  | 10.SCRM | | | | | | 6 |
|  | 11.BIM | | | | | | 16 |
|  | 12.ATB2 | | | | | | 1 |
|  | 13.ILR3 | | | | | | 13 |
|  | 14.PIF3 | | | | | | 4 |
|  | 15.ATR2 | | | | | | 2 |
|  | 16.TCP20 | | | | | | 14 |
|  | 17.HY5 | | | | | | 3 |
|  | 18.BIM1 | | | | | | 7 |
|  | 19.GLABRA 3 | | | | | | 1 |
| **Regulator** | | | | | | | 14 |
|  | 1.cAMP-binding proteins | | | | | | 3 |
|  | 2.regulator of gene silencing | | | | | | 2 |
|  | 3.ran1 | | | | | | 8 |
|  | 4.MYL9 | | | | | | 1 |
| **Oxidative Burst** | | | | | | | 288 |
|  | 1.Cu/Zn superoxide dismutase (SOD1) | | | | | | 31 |
|  | 2.Superoxide-generating NADPH oxidase heavy chain subunit A (noxA) | | | | | | 2 |
|  | 3.Superoxide-generating NADPH oxidase heavy chain subunit B (nox B) | | | | | | 2 |
|  | 4.copper chaperone for superoxide dismutase (Ccs) | | | | | | 1 |
|  | 5.superoxide dismutase | | | | | | 54 |
|  | 6.Superoxide dismutase [Mn] (SOD2) | | | | | | 16 |
|  | 7.superoxide dismutase, Fe-Mn family | | | | | | 22 |
|  | 8.Superoxide dismutase [Fe] (SOD B) | | | | | | 4 |
|  | 9.catalase (Cat ) | | | | | | 13 |
|  | 10.Catalase isozyme A | | | | | | 2 |
|  | 11.Catalase-peroxidase | | | | | | 6 |
|  | 12.Catalase-A | | | | | | 4 |
|  | 13.Peroxidase (PER70) | | | | | | 5 |
|  | 14.thioredoxin peroxidase | | | | | | 4 |
|  | 15.ascorbate peroxidase | | | | | | 22 |
|  | 16.cytochrome c peroxidase | | | | | | 5 |
|  | 17.glutathione peroxidase | | | | | | 24 |
|  | 18.Cationic peroxidase | | | | | | 10 |
|  | 19.NADPH oxidase | | | | | | 6 |
|  | 20.respiratory burst oxidase (RBOH) | | | | | | 48 |
|  | 21. rac GTPase activating protein | | | | | | 7 |
| **Programmed cell death protein** | | | | | | | 178 |
|  | 1.Programmed cell death protein 6 | | | | | | 1 |
|  | 2.caspase/ cysteine-type endopeptidase | | | | | | 6 |
|  | 3.Metacaspase | | | | | | 15 |
|  |  | | | Metacaspase-2 (AMC2) | | | 2 |
|  |  |  |  | AMC1 | | | 6 |
|  |  |  |  | AMC4 | | | 3 |
|  |  |  |  | AMC5 | | | 4 |
|  | 4.AtMC2 | | | | | | 2 |
|  | 5.MCA1 | | | | | | 2 |
|  | 6.Acin1 | | | | | | 2 |
|  | 7.defender against apoptotic death 1 | | | | | | 2 |
|  | 8.Bcl-2-associated athanogenes(BAGs) | | | | | | 8 |
|  |  | | Bcl-2-associated athanogene 1 | | | | 1 |
|  |  |  | Bcl-2-associated athanogene 3 | | | | 1 |
|  |  |  | Bcl-2-associated athanogene 6 | | | | 2 |
|  |  |  | Bcl-2-associated athanogene 7 | | | | 3 |
|  |  |  | BCL-2 binding anthanogene-1 | | | | 1 |
|  | 9.C-Myc-binding protein (MYCBP) | | | | | | 3 |
|  | 10.Putative apoptosis inhibitor ORF106 | | | | | | 1 |
|  | 11.Apoptosis-inducing factor 2 (aifm2) | | | | | | 4 |
|  | 12.apoptosis inhibitory 5 (API5) | | | | | | 3 |
|  | 13.Cell division cycle and apoptosis regulator protein 1(ccar1) | | | | | | 1 |
|  | 14.Apoptosis inhibitor 5 homolog (Aac11) | | | | | | 2 |
|  | 15.Rho GTPase-activating protein | | | | | | 10 |
|  | 16.AGD6 | | | | | | 5 |
|  | 17.AGD7 | | | | | | 7 |
|  | 18.AGD8 | | | | | | 7 |
|  | 19.AGD11 | | | | | | 16 |
|  | 20.gacA | | | | | | 4 |
|  | 21.GTPase (SAR1) | | | | | | 3 |
|  | 22.rac GTPase activating protein 1 | | | | | | 5 |
|  | 23.Ran-specific GTPase-activating protein 2 (YRB2) | | | | | | 1 |
|  | 24.Rab5/RabF-family small GTPase | | | | | | 2 |
|  | 25.Rab GTPase interacting factor | | | | | | 5 |
|  | 26.Ras GTPase-activating protein-binding protein 1 (G3bp1) | | | | | | 5 |
|  | 27.Mitochondrial Rho GTPase 1 (GEM1) | | | | | | 3 |
|  | 28.GPN-loop GTPase 2 | | | | | | 1 |
|  | 29.rho GTPase activator | | | | | | 4 |
|  | 30.GTPase-activating protein GYP7 | | | | | | 12 |
|  | 31.binding / small GTPase regulator (EMBRYO DEFECTIVE 2754) | | | | | | 1 |
|  | 32.dynamin GTPase | | | | | | 19 |
|  | 33. Dynamin-related proteins (DRP) | | | | | | 16 |
| **Pathogenesis-related** | | | | | | | 74 |
|  | Pathogenesis-related protein | | | | | | 33 |
|  | Thaumatin-Like Protein (TLP) | | | | | | 41 |
| [**Natural killer cell mediated cytotoxicity**](../../../../O:%5CF-pan%5C实验结果%5C杨静%5C测序转录组数据%5C转录组%5Cskn110325%5Cannotation%5CKEGG%5CAll-Unigene.fa_map%5Cmap04650.html) | | | | | | | **198** |
|  | 1.Rac-like GTP-binding protein ARAC5 | | | | | | 2 |
|  | 2.Ocs element-binding factor 1 | | | | | | 28 |
|  | 3.Ras-related C3 botulinum toxin substrate 3 | | | | | | 2 |
|  | 4.Calcineurin B-like protein 3 | | | | | | 6 |
|  | 5.GTP-binding protein rhoA | | | | | | 3 |
|  | 6.Transcription factor HBP-1a | | | | | | 14 |
|  | 7.Protein FD | | | | | | 3 |
|  | 8.Rho-related protein racB | | | | | | 1 |
|  | 9.Cell division control protein 42 | | | | | | 4 |
|  | 10.ABSCISIC ACID-INSENSITIVE 5-like protein 2 | | | | | | 4 |
|  | 11.Sulfated surface glycoprotein 185 | | | | | | 65 |
|  | 12.Molecular chaperone | | | | | | 66 |
|  |  | | | | HSP90 family (HtpG) | | 57 |
|  |  |  |  |  | Heat shock protein 90 | | 8 |
|  |  |  |  |  | Heat shock protein 82 | | 1 |
| **G-protein** | | | | | | | 124 |
|  | Ran/TC4 | | | | | | 12 |
|  | Ran1B | | | | | | 3 |
|  | Ran | | | | | | 2 |
|  | ERG | | | | | | 24 |
|  | YPTV3 | | | | | | 6 |
|  | YPT1 | | | | | | 5 |
|  | TYPA/BipA | | | | | | 2 |
|  | Spg1 | | | | | | 6 |
|  | Rab1-like small- | | | | | | 3 |
|  | Sar1 | | | | | | 8 |
|  | Sar1A | | | | | | 12 |
|  | RhoA | | | | | | 3 |
|  | lepA | | | | | | 5 |
|  | Yptm2 | | | | | | 11 |
|  | Eftud1 | | | | | | 1 |
|  | Rab6 | | | | | | 3 |
|  | Racdp | | | | | | 3 |
|  | rac5 | | | | | | 12 |
|  | RragC | | | | | | 1 |
|  | GTPbp4 | | | | | | 1 |
|  | Drg2 | | | | | | 1 |
| **plant horomone Biosythnesis and Signaling** | | | | | | | **643** |
|  | 1.Salicylie acid | | | | | | 210 |
|  |  | | | | phenylalanine ammonia lyase（PAL） | | 13 |
|  |  |  |  |  | isochorismate synthase(ICS) | | 4 |
|  |  |  |  |  | NPR1 | | 18 |
|  |  |  |  |  | MYB transcriptional factors | | 160 |
|  |  |  |  |  | EDS5(Enhanced disease susceptibility 5) | | 2 |
|  |  |  |  |  | EDS1 | | 1 |
|  |  |  |  |  | PAD4 | | 1 |
|  |  |  |  |  | NDR1 | | 10 |
|  |  |  |  |  | others | | 1 |
|  | 2.ethylene | | | | | | 262 |
|  |  | | | | ACC synthase(ACS) | | 1 |
|  |  |  |  |  | ACC oxidase(ACO) | | 2 |
|  |  |  |  |  | Others | | 259 |
|  | 3.jasmonic acid (JA) | | | | | | 98 |
|  | | | | | Lipoxygenase (LOX) | | 54 |
|  |  |  |  |  | Allene oxide synthase (AOS) | | 20 |
|  |  |  |  |  | Jasmonate ZIM-motif (JAZ) proteins | | 10 |
|  |  |  |  |  | Protein TIFY 10B | | 10 |
|  |  |  |  |  | Extracellular superoxide dismutase [Cu-Zn] 2 | | 1 |
|  |  |  |  |  | others | | 4 |
|  | 4.ABA | | | | | | 44 |
|  | | | | | ZE(zeaxanthin epoxidase) | | 6 |
|  |  |  |  |  | 9-cis-epoxycarotenoid dioxygenase（NCED） | | 7 |
|  |  |  |  |  | others | | 31 |
|  | 5. Brassinosteroid biosynthesis | | | | | | 39 |
|  | 6.NO | | | | | | 34 |
|  | | | | | Nitric oxide synthase(NOS) | | 6 |
|  |  |  |  |  | arginine decarboxylase(ADc) | | 16 |
|  |  |  |  |  | Arginase | | 4 |
|  |  |  |  |  | nitrate reductase (NR) | | 8 |
| Flavonoid | | | | | | | 377 |
|  | chalcone synthase (CHS) | | | | | | 21 |
|  | chalcone isomerase (CHI) | | | | | | 3 |
|  | shikimate *O-*hydroxycinnamoyltransferase | | | | | | 59 |
|  | nthocyanidin reductase（AtANR） | | | | | | 1 |
|  | PAL | | | | | | 13 |
|  | others | | | | | | 280 |
| Cell wall modification | | | | | | | 574 |
|  | 3-deoxy--D-arabino-heptulosonate 7-phosphate synthase (DAHPS) | | | | | | 4 |
|  | 4-coumarate--CoA ligase (4CL) | | | | | | 49 |
|  | polyphenol oxidase (PPO) | | | | | | 11 |
|  | Glutathione-S-transferase (GST) | | | | | | 47 |
|  | galactosidase | | | | | | 61 |
|  | extension | | | | | | 39 |
|  | UDP-glucuronic acid decarboxylase | | | | | | 32 |
|  | Cellulose synthase | | | | | | 89 |
|  | Callose synthase | | | | | | 53 |
|  | PDCB3 (PLASMODESMATA CALLOSE-BINDING PROTEIN 3) | | | | | | 3 |
|  | beta-1,3-glucanase | | | | | | 21 |
|  | Glucan endo-1,3-beta-glucosidase (glc) | | | | | | 106 |
|  | Exo-beta-1,3-glucanase | | | | | | 21 |
|  | Caffeic acid 3-O-methyltransferase (COMT) | | | | | | 38 |
