# Supplementary material for: Transcriptome profiling of resistant and susceptible Cavendish banana roots following inoculation with Fusarium oxysporum f. sp. cubense tropical race 4
Source: BMC Genomics. 2012 Aug 5;13:374. doi: 10.1186/1471-2164-13-374 (PMC3473311; doi:10.1186/1471-2164-13-374)
Supplement: Additional file 2 — Table S2.List of metabolic pathways related to host defense in banana following inoculation with Fusarium oxysporum f. sp. cubense tropical race 4 [7,13,26-70]. [file 1471-2164-13-374-S2.doc]

Additional file 2, Table S2

| Pathway | Differentially expressed genes | Functions | References |
| --- | --- | --- | --- |
| Perception of PAMPs by PRRs | Chitin elicitor-binding protein (CEBiP) | Perception and transduction of the chitin elicitor signal | [6] |
|  | Chitin elicitor receptor kinase (CERK1) | Chitin elicitor signaling | [6] |
|  | Elicitor-responsive proteins (ERG) | Plant defense signaling | [7] |
|  | Proline-rich Extensin-like Receptor Kinases (PERKs) | Perception of PAMPs and induce defense responses | [8] |
|  | BRI1-Associated receptor Kinase 1 (BAK1) | Perception of PAMPs and induce defense responses | [9] |
|  | Flagellin-sensing2-like genes (FLS2) | Perception of pathogen elicitors | [10] |
|  | Somatic embryogenesis receptor-like kinases (SERKs) | Implication in plant immune responses to pathogen attack | [11] |
|  | Plant receptor-like kinases (RLKs) | Perception of PAMPs and induce defense responses | [12] |
|  | Mitogen-activated protein kinase (MAPK) | Downstream components in PTI | [13] |
| Effector-  triggered immunity (ETI) | CC-NB-LRR protein (RPM1) | Recognition of the altered status and initiates a defense-signaling response: hypersensitive response (HR) | [14] |
|  | [Disease resistance protein](http://www.google.com.hk/url?sa=t&rct=j&q=RPS2&source=web&cd=22&ved=0CCkQFjABOBQ&url=http%3A%2F%2Fwww.wikigenes.org%2Fe%2Fgene%2Fe%2F828715.html&ei=RcqdT8FhhqSIB-7Y2M4O&usg=AFQjCNEHbXOMmg60520fHg_GYLy4i_u7Rw) (RPS2) | Specifically recognizes effector protein from pathogen | [15] |
|  | RPM1 Interacting Protein 4 (RIN4) | Negatively regulates disease resistance mediated by RPS2 | [15] |
| Ion Fluxes | Plant cyclic nucleotide gated channels (CNGCs) | Facilitates Ca^2+^ uptake into the cytosol in response to PAMP | [16] |
|  | Calmodulin (CaM) | Calcium signal transducer | [17] |
|  | CaM-related proteins | Calcium signal transducer | [17] |
|  | Calmodulin-like protein (CML) | Calcium signal transducer | [17] |
|  | Calcineurin B-like proteins (CBL) | Decoding of calcium transients | [18] |
| Transcription factors (TFs) | WRKY | Regulation of  signaling and transcriptional reprogramming associated with plant defense responses | [19] |
|  | BHLH | Regulation of  signaling and transcriptional reprogramming associated with plant defense responses | [20] |
|  | ERF | Binding ethylene- responsive-element | [21] |
|  | Histone promoter-binding protein ( HBP )-1a | Plays a negative regulation role in the defense response to Foc TR4 attack | [22] |
| Oxidative burst | NADPH oxidase or respiratory burst oxidase | Generation of [superoxide](http://en.wikipedia.org/wiki/Superoxide) | [23] |
|  | Catalase | Decomposition of [hydrogen peroxide](http://en.wikipedia.org/wiki/Hydrogen_peroxide) to [water](http://en.wikipedia.org/wiki/Water) and [oxygen](http://en.wikipedia.org/wiki/Oxygen) | [24] |
|  | Ascorbate peroxidase | Detoxify [peroxides](http://en.wikipedia.org/wiki/Peroxides) | [25] |
|  | Thioredoxin peroxidases | Reduction of various peroxides | [26] |
|  | Glutathione peroxidases (GPXs) | Reduction of H_2_O_2_, organic hydroperoxidases, and lipid [peroxides](http://en.wikipedia.org/wiki/Organic_peroxide) | [27] |
|  | Cationic peroxidases | Causes a disease resistance response | [28] |
|  | Protein disulfide-isomerase (PDI) | Ubiquitous redox protein | [29] |
| Pathogenesis-related (PR) proteins | PR1 protein | Confers resistance to pathogen and hallmarks of defense pathways | [30-31] |
|  | PR1C | Confers resistance to pathogen and hallmarks of defense pathways | [30-31] |
|  | Beta-1,3-glucanase-like genes (PR2 homologs) | Lyses cell walls of fungal pathogens | [30-31] |
|  | Chitinase (PR3 and 8 homologs) | Lyses cell walls of fungal pathogens | [32-33] |
|  | Thaumatin-Like Protein (PR5) | Inhibition of hyphal growth and sporulation by various [fungi](http://en.wikipedia.org/wiki/Fungi) | [34] |
| Programmed cell death (PCD) | BAG-like genes: BCL-2-associated athanogenes | Suppresses [apoptosis](javascript:if(window.name=='') {{ window.location.href='./nil'; }} else {{ NPEml('MeSH',12305); }}) | [35] |
|  | Dynamin-related proteins (DRP) | Key regulators of PCD | [36] |
|  | Apoptosis Inducing Factor (AIF) | Chromatin condensation and DNA degradation | [37] |
| Plant hormones | Nitric oxide synthase | Catalyzes arginine to produce nitric oxide | [38] |
|  | Non-expression of PR gene 1 (NPR1) | Regulatory component in SA signaling | [39] |
|  | Pathogen-inducible salicylic acid glucosyltransferase (SAG) | Involved in the early disease response and the accumulation of glucosyl SA during pathogenesis | [40] |
|  | Phenylalanine ammonia lyase (PAL) | Key enzyme in SA biosynthesis | [41]. |
|  | Isochorismate synthase (ICS) | Key enzyme in SA biosynthesis | [41] |
|  | Lipoxygenase (LOX) | Key enzyme in jasmonic acid (JA) biosynthesis | [42-43] |
|  | Allene oxide synthase (AOS) | Key enzyme in jasmonic acid biosynthesis | [42-43] |
|  | Jasmonate ZIM-motif (JAZ) proteins (TIFY10B) | JA signaling | [44] |
|  | 1-aminocyclopropane-1-carboxylic acid oxidase (ACO) | Key enzyme in Ethylene biosynthesis | [45] |
| Cell wall modification | 3-deoxy-D-arabino-heptulosonate 7-phosphate synthase | Biosynthesis of derived secondary metabolites | [46] |
|  | 4-coumarate-CoA ligase | Phenylpropanoid metabolism | [47] |
|  | Polyphenol oxidase | Oxidation of phenol compounds | [48] |
|  | Glutathione-S-transferase | Conjugation of electrophilic molecules to glutathione (GSH) | [49] |
|  | Caffeic acid 3-O-methyltransferase (COMT) | Lignin biosynthesis | [50] |
|  | Extension | Inhibition of pathogen invasion | [51] |
|  | Cellulose synthase | Callose synthesis | [52] |
