# Supplementary material for: Transcriptome profiling of resistant and susceptible Cavendish banana roots following inoculation with Fusarium oxysporum f. sp. cubense tropical race 4
Source: BMC Genomics. 2012 Aug 5;13:374. doi: 10.1186/1471-2164-13-374 (PMC3473311; doi:10.1186/1471-2164-13-374)
Supplement: Additional file 3 — Table S3.Expression of unigenes in ‘Brazilian’ (susceptible wild-type) and cv ‘Nongke No 1’ (resistant mutant) bananas following inoculation with Fusarium oxysporum f. sp. cubense tropical race 4. [file 1471-2164-13-374-S3.doc]

Additional file 3, Table S3：All of the unigenes used in the result section.

|  | Unigene ID | annotation | Expression levels (rpkm) | | | | | |
| --- | --- | --- | --- | --- | --- | --- | --- | --- |
|  |  |  | BK | B1 | B2 | NKCK | NK1 | NK2 |
| CEBiP | 13093 | Chitin elicitor-binding protein [Oryza sativa subsp. Japonica] | 85.56 | 144.58 | 145.16 | 57.16 | 136.43 | 245.23 |
| Elicitor-responsive protein | 84621 | Elicitor-responsive protein 1 [Oryza sativa subsp. Japonica] | 9.00 | 20.20 | 35.47 | 0.39 | 19.59 | 115.95 |
|  | 4188 | Elicitor-responsive protein 1 [Oryza sativa subsp. japonica ] | 16.55 | 65.87 | 108.57 | 0.71 | 61.36 | 357.77 |
| Chitin elicitor receptor kinase (CERK1) | 5486 | Chitin elicitor receptor kinase (CERK1) | 14.22 | 30.75 | 21.00 | 7.70 | 27.16 | 47.07 |
|  | 44022 | Chitin elicitor receptor kinase (CERK1) | 108.51 | 161.20 | 116.33 | 53.23 | 149.79 | 169.80 |
|  | 32231 | Chitin elicitor receptor kinase (CERK1) | 81.37 | 120.54 | 97.68 | 42.01 | 123.75 | 135.43 |
|  | 29711 | Chitin elicitor receptor kinase (CERK1) | 77.39 | 91.05 | 105.77 | 30.29 | 117.78 | 159.05 |
|  | 14228 | Chitin elicitor receptor kinase (CERK1) | 164.52 | 232.42 | 201.23 | 62.17 | 270.06 | 234.94 |
| PERK10 | 18760 | Proline-rich receptor-like protein kinase PERK10 [Arabidopsis thaliana ] | 204.99 | 234.10 | 145.96 | 306.17 | 269.37 | 349.94 |
|  | 17067 | Proline-rich receptor-like protein kinase PERK10 [Arabidopsis thaliana ] | 40.97 | 90.92 | 63.00 | 28.48 | 114.17 | 123.71 |
| PERK13 | 23324 | Proline-rich receptor-like protein kinase PERK13 [Arabidopsis thaliana ] | 70.28 | 125.11 | 81.56 | 96.70 | 157.00 | 172.59 |
| BAK1 (BRI1-Associated receptor Kinase 1) | 7884 | BRASSINOSTEROID INSENSITIVE 1-associated receptor kinase 1 [Arabidopsis thaliana ] | 358.03 | 216.23 | 405.47 | 328.99 | 179.54 | 111.62 |
|  | 49719 | BRASSINOSTEROID INSENSITIVE 1-associated receptor kinase 1 [Arabidopsis thaliana ] | - | 63.66 | - | - | - | - |
|  | 43526 | Probable receptor-like protein kinase At1g11050 [Arabidopsis thaliana] | 21.92 | 59.85 | 33.34 | 13.74 | 55.31 | 88.50 |
|  | 40337 | Probable LRR receptor-like serine/threonine-protein kinase At5g10290 [Arabidopsis thaliana] | 36.65 | 50.28 | 34.26 | 44.18 | 70.26 | 73.00 |
| FLS2 | 19603 | LRR receptor-like serine/threonine-protein kinase FLS2 [Arabidopsis thaliana ] | 19.02 | 60.17 | 18.53 | 10.25 | 53.58 | 68.18 |
| SERK1 | 7885 | Somatic embryogenesis receptor kinase 1 [Arabidopsis thaliana ] | 270.90 | 177.61 | 244.78 | 318.86 | 171.83 | 151.06 |
|  | 75864 | Somatic embryogenesis receptor kinase 1 [Arabidopsis thaliana ] | 661.53 | 295.85 | 643.23 | 468.43 | 321.32 | 162.87 |
| MPK1 | 61915 | Mitogen-activated protein kinase 1 [Oryza sativa subsp. japonica ] | - | 13.18 | - | - | - | - |
| MPK2 | 40623 | Mitogen-activated protein kinase 2 [Oryza sativa subsp. japonica ] | 63.26 | 34.64 | 52.49 | 75.01 | 34.32 | 24.38 |
| MPK5 | 8594 | Mitogen-activated protein kinase 5 [Oryza sativa subsp. japonica ] | 24.65 | 54.64 | 29.85 | 35.55 | 50.88 | 77.23 |
| MPK6 | 15683 | Mitogen-activated protein kinase 6 [Oryza sativa subsp. japonica ] | 97.95 | 47.83 | 72.26 | 86.67 | 58.12 | 24.91 |
| MPK10 | 36751 | Mitogen-activated protein kinase 10 [Oryza sativa subsp. japonica ] | 31.57 | 43.42 | 22.58 | 90.42 | 57.61 | 36.41 |
| MPK12 | 5862 | Mitogen-activated protein kinase 12 [Oryza sativa subsp. japonica ] | 163.51 | 176.28 | 157.77 | 170.88 | 183.99 | 218.84 |
| Mitogen-activated protein kinase kinase 4 | 63073 | Mitogen-activated protein kinase kinase 4 [Arabidopsis thaliana ] | - | 50.43 | - | - | - | - |
| Mitogen-activated protein kinase kinase 5 | 47292 | Mitogen-activated protein kinase kinase 5 [Arabidopsis thaliana ] | - | 22.71 | - | - | - | - |
|  | 4515 | Mitogen-activated protein kinase kinase 5 | 2.64 | 0.99 | 3.28 | 2.92 | - | 0.71 |
| Mitogen-activated protein kinase kinase kinase 2(MEKK2) | 496 | Mitogen-activated protein kinase kinase kinase 2 [Arabidopsis thaliana ] | 64.69 | 111.19 | 51.79 | 84.54 | 90.41 | 130.16 |
|  | 38455 | Mitogen-activated protein kinase kinase kinase 2 [Arabidopsis thaliana ] | 72.36 | 95.96 | 55.70 | 127.72 | 92.29 | 84.10 |
| mitogen-activated protein kinase kinase 4 | 63073 | Mitogen-activated protein kinase kinase 4 [Arabidopsis thaliana ] | - | 50.43 | - | - | - | - |
| mitogen-activated protein kinase kinase 5 | 47292 | Mitogen-activated protein kinase kinase 5 [Arabidopsis thaliana ] | - | 22.71 | - | - | - | - |
| PBS1 | 7920 | Serine/threonine-protein kinase PBS1 [Arabidopsis thaliana ] | 12.86 | 5.29 | 6.91 | 26.04 | 8.89 | 5.28 |
|  | 75105 | Serine/threonine-protein kinase PBS1 [Arabidopsis thaliana ] | 1.19 | 0.67 | 2.67 | 11.84 | 4.07 | 0.96 |
|  | 7463 | Serine/threonine-protein kinase PBS1 [Arabidopsis thaliana ] | 45.65 | 44.67 | 32.43 | 85.03 | 62.35 | 27.49 |
|  | 5647 | Serine/threonine-protein kinase PBS1 [Arabidopsis thaliana ] | 45.39 | 45.36 | 31.26 | 52.12 | 46.32 | 31.93 |
| RPS5 | 29504 | 40S ribosomal protein S5-1 [Arabidopsis thaliana] | 0.56 | 6.49 | 22.04 | 0.35 | 2.98 | 7.25 |
| RIN4 | 36589 | RPM1-interacting protein 4 [Arabidopsis thaliana ] | 26.65 | 11.55 | 15.98 | 62.83 | 13.55 | 14.11 |
|  | 16392 | RPM1-interacting protein 4 [Arabidopsis thaliana ] | 9.07 | 4.73 | 8.41 | 15.27 | 6.12 | 2.28 |
| RPS2 | 29749 | Disease resistance protein RPS2 | 5.51 | 1.17 | 4.01 | 7.56 | 4.42 | 1.93 |
| RPM1 | 34343 | Disease resistance protein RPM1 [Arabidopsis thaliana ] | 112.70 | 11.69 | 19.52 | 165.69 | 16.59 | 2.95 |
|  | 16450 | Disease resistance protein RPM1 [Arabidopsis thaliana ] | 69.56 | 7.67 | 11.74 | 97.53 | 8.64 | 0.90 |
|  | 30247 | disease resistance protein RPM1 | 42.95 | 5.36 | 7.98 | 60.19 | 6.41 | 0.45 |
| interleukin-1 receptor-associated kinase (IRAK) | 8303 | interleukin-1 receptor-associated kinase 4 | 39.63 | 22.01 | 25.45 | 69.68 | 26.55 | 13.02 |
|  | 31479 | interleukin-1 receptor-associated kinase 4 | 36.37 | 111.34 | 43.60 | 12.55 | 122.41 | 95.71 |
|  | 25972 | interleukin-1 receptor-associated kinase 4 | 28.46 | 56.72 | 37.39 | 23.46 | 61.58 | 89.63 |
|  | 2231 | interleukin-1 receptor-associated kinase 4 | 111.73 | 69.97 | 94.13 | 142.74 | 76.84 | 89.41 |
|  | 10478 | interleukin-1 receptor-associated kinase 4 | 339.51 | 332.51 | 243.41 | 223.69 | 351.80 | 230.13 |
| Calmodulin | 62249 | Calmodulin [Capsicum annuum] | - | 11.12 | - | - | - | - |
|  | 60227 | Calmodulin [Capsicum annuum] | - | 16.19 | - | - | - | - |
|  | 48035 | Calmodulin [Lilium longiflorum] | - | 29.86 | - | - | - | - |
|  | 57715 | Calmodulin [Medicago sativa ] | - | 10.15 | - | - | - | - |
|  | 23270 | Calmodulin [Pythium splendens] | 1.62 | 6.06 | 2.15 | 5.12 | 5.33 | 25.65 |
|  | 60660 | Calmodulin [Saccharina japonica ] | 0.40 | 21.32 | 21.75 | - | 0.46 | - |
|  | 12131 | Calmodulin [Spinacia oleracea ] | 30.53 | 33.88 | 47.76 | 9.32 | 13.61 | 27.30 |
|  | 21697 | Calmodulin [Xenopus laevis] | 1.02 | 8.04 | 6.12 | - | 1.17 | 37.33 |
|  | 12056 | calmoduline [Phaeodactylum tricornutum CCAP 1055/1] | 0.23 | 1.78 | 4.07 | - | 7.77 | 11.77 |
|  | 3777 | calmodulin [Heterocapsa triquetra] | 0.67 | 5.67 | 0.50 | 1.92 | 4.86 | 18.90 |
| Calmodulin-like protein | 7020 | Calmodulin-like protein 11 [Arabidopsis thaliana] | 15.44 | 14.90 | 31.40 | 0.59 | 13.03 | 42.03 |
|  | 20081 | Calmodulin-like protein 11 [Arabidopsis thaliana ] | 12.93 | 20.01 | 18.46 | 3.72 | 35.53 | 35.56 |
| Calcineurin B-like protein (CBL) | 29623 | Calcineurin B-like protein 3 [Oryza sativa subsp. japonica ] | 70.61 | 69.79 | 91.54 | 45.27 | 52.23 | 50.77 |
|  | 2185 | Calcineurin B-like protein 3 [Oryza sativa subsp. japonica ] | 22.81 | 11.08 | 14.25 | 35.84 | 20.19 | 13.24 |
|  | 19241 | Calcineurin B-like protein 3 [Oryza sativa subsp. japonica ] | 70.07 | 64.06 | 88.58 | 58.24 | 56.03 | 55.01 |
|  | 15742 | Calcineurin B-like protein 3 [Oryza sativa subsp. japonica ] | 30.38 | 23.51 | 19.74 | 46.70 | 26.76 | 14.94 |
| CIPK11 | 63320 | CBL-interacting serine/threonine-protein kinase 11 [Arabidopsis thaliana ] | - | 18.88 | - | - | - | - |
| CIPK14 | 63786 | CBL-interacting serine/threonine-protein kinase 14 [Arabidopsis thaliana ] | - | 37.73 | - | - | - | - |
| CIPK20 | 17700 | CBL-interacting serine/threonine-protein kinase 20 [Arabidopsis thaliana ] | 74.89 | 58.59 | 112.00 | 36.60 | 16.57 | 31.62 |
| CNGC1 | 34044 | Cyclic nucleotide-gated ion channel 1 [Arabidopsis thaliana ] | 33.36 | 61.56 | 27.67 | 31.87 | 64.44 | 64.70 |
| CNGC5 | 36624 | Probable cyclic nucleotide-gated ion channel 5 [Arabidopsis thaliana ] | 68.12 | 122.73 | 56.92 | 47.14 | 101.49 | 91.40 |
| CNGC6 | 34288 | Probable cyclic nucleotide-gated ion channel 6 [Arabidopsis thaliana ] | 60.09 | 111.81 | 41.68 | 49.20 | 89.65 | 68.86 |
| voltage-gated potassium channel | 37754 | Probable voltage-gated potassium channel subunit beta [Oryza sativa subsp. Japonica] | 113.84 | 129.12 | 166.66 | 51.82 | 125.76 | 125.02 |
|  | 13869 | Probable voltage-gated potassium channel subunit beta [Oryza sativa subsp. Japonica] | 35.03 | 37.70 | 39.43 | 18.16 | 42.02 | 52.09 |
| Potassium channel AKT1 | 22369 | Potassium channel AKT1 [Oryza sativa subsp. Japonica] | 12.76 | 47.78 | 17.07 | 11.46 | 31.56 | 35.90 |
|  | 16753 | Potassium channel AKT1 [Oryza sativa subsp. Japonica] | 27.20 | 68.95 | 25.81 | 23.71 | 53.43 | 54.62 |
|  | 20103 | Potassium channel AKT2/3 [Arabidopsis thaliana ] | 13.43 | 2.42 | 1.33 | 37.49 | 6.50 | 0.62 |
|  | 34386 | Potassium channel SKOR [Arabidopsis thaliana ] | 3.42 | 22.55 | 7.85 | 4.55 | 14.97 | 7.21 |
| Chloride channel protein CLC-b | 18382 | Chloride channel protein CLC-b [Arabidopsis thaliana] | 32.70 | 10.96 | 15.33 | 29.17 | 16.70 | 10.28 |
| ATP synthase subunit delta', mitochondrial | 3562 | ATP synthase subunit delta', mitochondrial [Ipomoea batatas] | 93.48 | 70.52 | 104.74 | 77.84 | 74.41 | 41.40 |
|  | 21825 | ATP synthase subunit delta', mitochondrial [Ipomoea batatas] | 125.81 | 100.20 | 195.41 | 129.17 | 116.59 | 133.12 |
|  | 10881 | ATP synthase subunit delta', mitochondrial [Ipomoea batatas] | 50.55 | 35.69 | 60.97 | 28.23 | 24.65 | 6.61 |
| WRKY6 | 43914 | WRKY transcription factor 6 [Arabidopsis thaliana ] | 49.09 | 68.78 | 47.16 | 42.15 | 67.44 | 112.06 |
|  | 43141 | WRKY transcription factor 6 [Arabidopsis thaliana ] | 46.91 | 86.70 | 86.41 | 23.88 | 73.09 | 121.44 |
|  | 18054 | WRKY transcription factor 6 [Arabidopsis thaliana ] | 175.74 | 375.71 | 208.65 | 122.80 | 354.26 | 570.61 |
| WRKY26 | 45076 | Probable WRKY transcription factor 26 [Arabidopsis thaliana ] | 74.17 | 156.41 | 96.67 | 74.38 | 164.39 | 305.63 |
|  | 10993 | Probable WRKY transcription factor 26 [Arabidopsis thaliana ] | 102.34 | 185.39 | 109.04 | 39.17 | 132.28 | 290.39 |
| WRKY28 | 36109 | Probable WRKY transcription factor 28 [Arabidopsis thaliana ] | 14.49 | 11.16 | 27.22 | 26.06 | 25.57 | 55.71 |
| WRKY31 | 2655 | Probable WRKY transcription factor 31 [Arabidopsis thaliana ] | 100.91 | 183.31 | 163.74 | 167.01 | 238.68 | 478.48 |
|  | 15847 | Probable WRKY transcription factor 31 [Arabidopsis thaliana ] | 77.69 | 136.17 | 104.36 | 119.99 | 166.99 | 308.54 |
| WRKY33 | 23111 | Probable WRKY transcription factor 33 [Arabidopsis thaliana ] | 100.31 | 178.32 | 99.10 | 37.03 | 116.23 | 277.27 |
|  | 1850 | Probable WRKY transcription factor 33 [Arabidopsis thaliana ] | 17.07 | 19.64 | 12.13 | 4.86 | 11.20 | 33.89 |
| WRKY33 | 10155 | Probable WRKY transcription factor 33 [Arabidopsis thaliana ] | 167.88 | 341.29 | 196.93 | 146.17 | 346.43 | 506.63 |
| WRKY40 | 35756 | Probable WRKY transcription factor 40 [Arabidopsis thaliana ] | 57.89 | 194.03 | 172.54 | 9.28 | 191.13 | 197.52 |
| WRKY60 | 1865 | Probable WRKY transcription factor 60 [Arabidopsis thaliana ] | 31.08 | 82.49 | 77.04 | 3.91 | 74.63 | 119.92 |
| WRKY65 | 40362 | Probable WRKY transcription factor 65 [Arabidopsis thaliana ] | 26.14 | 50.87 | 29.65 | 24.39 | 43.99 | 111.91 |
|  | 16313 | Probable WRKY transcription factor 65 [Arabidopsis thaliana ] | 15.81 | 43.79 | 32.03 | 16.69 | 40.14 | 102.05 |
| WRKY72 | 7854 | Probable WRKY transcription factor 72 [Arabidopsis thaliana ] | 59.21 | 151.91 | 69.30 | 82.27 | 119.86 | 148.25 |
|  | 58391 | Probable WRKY transcription factor 72 [Arabidopsis thaliana ] | 104.18 | 325.82 | 125.37 | 191.22 | 277.48 | 369.04 |
| WRKY72 | 15850 | Probable WRKY transcription factor 72 [Arabidopsis thaliana ] | 117.72 | 308.55 | 86.26 | 152.99 | 240.37 | 321.10 |
| WRKY75 | 37610 | Probable WRKY transcription factor 75 [Arabidopsis thaliana ] | 38.74 | 24.09 | 74.41 | - | 36.18 | 6.15 |
| BHLH18 | 23748 | Transcription factor bHLH18 [Arabidopsis thaliana ] | 8.97 | 1.12 | 1.92 | 29.18 | 1.30 | 0.23 |
| BHLH25 | 15237 | Transcription factor bHLH25 [Arabidopsis thaliana ] | 29.93 | 10.62 | 22.91 | 22.30 | 13.77 | 7.09 |
| BHLH30 | 36152 | Transcription factor bHLH30 [Arabidopsis thaliana ] | 22.66 | 26.15 | 14.70 | 41.57 | 17.43 | 15.41 |
|  | 2606 | Transcription factor bHLH30 [Arabidopsis thaliana ] | 28.79 | 10.94 | 19.39 | 39.86 | 15.40 | 10.58 |
| BHLH35 | 34524 | Transcription factor bHLH35 [Arabidopsis thaliana ] | 1.81 | 1.02 | - | 18.45 | 0.52 | - |
|  | 3086 | Transcription factor bHLH35 [Arabidopsis thaliana ] | 2.69 | 0.18 | 0.71 | 20.13 | 0.54 | 0.26 |
| BIM (BES1-interacting Myc-like protein)1 | 21596 | Transcription factor BIM1 [Arabidopsis thaliana ] | 28.20 | 21.15 | 11.74 | 41.71 | 22.69 | 20.89 |
| BIM2 | 9411 | Transcription factor BIM2 [Arabidopsis thaliana ] | 32.58 | 14.30 | 27.36 | 49.47 | 22.74 | 12.68 |
| BIM2 | 29736 | Transcription factor BIM2 [Arabidopsis thaliana ] | 18.85 | 13.12 | 16.31 | 38.13 | 19.73 | 13.13 |
| Ethylene-responsive transcription factor (ERF) | 69445 | Ethylene-responsive transcription factor 1 [Oryza sativa subsp. japonica ] | 302.96 | 300.34 | 400.13 | 242.19 | 268.71 | 144.52 |
|  | 2419 | Ethylene-responsive transcription factor 1 [Oryza sativa subsp. japonica ] | 165.81 | 196.18 | 106.71 | 265.85 | 193.07 | 152.49 |
|  | 20980 | Ethylene-responsive transcription factor 1 [Oryza sativa subsp. japonica ] | 198.32 | 69.37 | 87.58 | 171.77 | 60.24 | 44.83 |
|  | 18972 | Ethylene-responsive transcription factor 1 [Oryza sativa subsp. japonica ] | 252.57 | 306.48 | 223.68 | 441.04 | 311.86 | 277.22 |
|  | 1251 | Ethylene-responsive transcription factor 1 [Oryza sativa subsp. japonica ] | 95.15 | 245.61 | 117.46 | 113.99 | 264.57 | 325.64 |
|  | 49282 | Ethylene-responsive transcription factor 1A [Arabidopsis thaliana ] | - | 34.40 | - | - | - | - |
|  | 76315 | Ethylene-responsive transcription factor 1B [Arabidopsis thaliana ] | 46.35 | 111.87 | 36.39 | 6.60 | 32.39 | 51.64 |
|  | 63761 | Ethylene-responsive transcription factor 1B [Arabidopsis thaliana ] | - | 30.89 | - | - | - | - |
|  | 49361 | Ethylene-responsive transcription factor 1B [Arabidopsis thaliana ] | - | 26.54 | - | - | - | - |
|  | 28653 | Ethylene-responsive transcription factor 1B [Arabidopsis thaliana ] | 53.16 | 115.89 | 56.93 | 10.74 | 38.73 | 71.14 |
|  | 23667 | Ethylene-responsive transcription factor 1B [Arabidopsis thaliana ] | 73.53 | 118.81 | 71.14 | 8.44 | 39.35 | 59.13 |
|  | 30933 | Ethylene-responsive transcription factor 11 [Arabidopsis thaliana ] | 104.90 | 15.34 | 18.01 | 72.49 | 10.04 | 43.90 |
|  | 9226 | Ethylene-responsive transcription factor 1 [Oryza sativa subsp. japonica ] | 179.63 | 295.33 | 220.31 | 215.64 | 245.27 | 380.19 |
| Transcription factor HBP(histone promoter-binding protein/0-1a | 37206 | Transcription factor HBP-1a [Triticum aestivum ] | 36.12 | 27.33 | 26.40 | 66.22 | 36.34 | 22.69 |
|  | 36291 | Transcription factor HBP-1a [Triticum aestivum ] | 24.23 | 18.00 | 19.29 | 40.19 | 28.84 | 15.82 |
|  | 31401 | Transcription factor HBP-1a [Triticum aestivum] | 15.68 | 8.80 | 7.26 | 26.33 | 15.05 | 7.66 |
|  | 2431 | Transcription factor HBP-1a [Triticum aestivum] | 19.88 | 13.92 | 10.67 | 25.95 | 17.73 | 10.74 |
|  | 19556 | Transcription factor HBP-1a [Triticum aestivum] | 16.63 | 8.80 | 7.98 | 36.90 | 15.39 | 7.63 |
| Cationic peroxidase | 4903 | Cationic peroxidase 1 [Arachis hypogaea ] | 116.55 | 93.15 | 123.53 | 40.07 | 99.39 | 15.89 |
|  | 38286 | Cationic peroxidase 1 [Arachis hypogaea ] | 39.06 | 109.94 | 51.33 | 7.68 | 91.03 | 67.82 |
|  | 63769 | Cationic peroxidase 2 [Arachis hypogaea ] | - | 62.66 | - | - | - | - |
|  | 48489 | Cationic peroxidase 2 [Arachis hypogaea ] | - | 23.68 | - | - | - | - |
| Thioredoxin peroxidase | 33231 | thioredoxin peroxidase [Elaeis guineensis] | 73.42 | 48.90 | 61.05 | 97.46 | 45.94 | 44.84 |
|  | 11757 | thioredoxin peroxidase [Elaeis guineensis] | 30.68 | 17.84 | 31.55 | 38.46 | 16.56 | 9.32 |
| Glutathione peroxidases (GPXs) | 82235 | Probable phospholipid hydroperoxide glutathione peroxidase [Mesembryanthemum crystallinum ] | 164.16 | 94.26 | 202.21 | 153.02 | 100.66 | 73.03 |
|  | 42581 | Probable phospholipid hydroperoxide glutathione peroxidase 6, mitochondrial [Arabidopsis thaliana ] | 75.50 | 43.38 | 62.50 | 67.35 | 56.39 | 24.76 |
|  | 41775 | Probable phospholipid hydroperoxide glutathione peroxidase 6, mitochondrial [Arabidopsis thaliana ] | 84.43 | 85.86 | 104.90 | 135.79 | 113.69 | 94.63 |
|  | 34202 | Probable phospholipid hydroperoxide glutathione peroxidase 6, mitochondrial [Arabidopsis thaliana ] | 75.39 | 67.35 | 87.40 | 93.87 | 89.77 | 56.18 |
| ascorbate peroxidases | 63766 | L-ascorbate peroxidase 3, peroxisomal [Arabidopsis thaliana ] | - | 81.10 | - | - | - | - |
|  | 63744 | L-ascorbate peroxidase 3, peroxisomal [Arabidopsis thaliana ] | - | 321.48 | - | - | - | - |
|  | 72605 | L-ascorbate peroxidase, cytosolic [Pisum sativum ] | 241.79 | 97.14 | 216.95 | 166.59 | 95.08 | 83.51 |
|  | 63279 | L-ascorbate peroxidase, cytosolic [Pisum sativum ] | - | 25.67 | - | - | - | - |
|  | 23508 | L-ascorbate peroxidase, cytosolic [Pisum sativum ] | 80.63 | 23.03 | 78.56 | 81.69 | 33.54 | 16.52 |
|  | 22416 | L-ascorbate peroxidase, cytosolic [Pisum sativum ] | 184.94 | 94.18 | 183.66 | 110.58 | 57.29 | 46.57 |
|  | 15558 | Probable L-ascorbate peroxidase 4 [Oryza sativa subsp. Japonica] | 102.31 | 75.83 | 109.84 | 175.76 | 109.47 | 60.97 |
| catalase | 8891 | Catalase isozyme 1 [Gossypium hirsutum] | 331.08 | 503.86 | 296.08 | 423.95 | 360.20 | 394.80 |
|  | 8722 | Catalase [Soldanella alpina] | 82.47 | 152.23 | 95.06 | 96.42 | 105.63 | 130.07 |
|  | 63913 | Catalase [Ipomoea batatas] | 0.16 | 282.01 | - | - | 0.09 | - |
|  | 63062 | Catalase isozyme 3 [Nicotiana plumbaginifolia] | 109.26 | 168.34 | 92.72 | 146.25 | 132.91 | 141.78 |
|  | 37659 | Catalase isozyme 2 [Hordeum vulgare] | 203.86 | 166.31 | 91.40 | 416.12 | 260.79 | 126.62 |
|  | 20259 | Catalase isozyme 1 [Gossypium hirsutum ] | 383.95 | 561.62 | 355.17 | 496.76 | 426.24 | 484.25 |
| RBOHB | 27163 | Respiratory burst oxidase homolog protein B [Solanum tuberosum ] | 36.63 | 84.02 | 40.25 | 61.03 | 100.10 | 137.03 |
|  | 25706 | Respiratory burst oxidase homolog protein B [Solanum tuberosum ] | 49.51 | 96.60 | 49.37 | 84.99 | 115.65 | 171.92 |
|  | 18553 | Respiratory burst oxidase homolog protein B [Solanum tuberosum ] | 35.04 | 53.38 | 32.94 | 17.57 | 48.54 | 55.16 |
| RBOHC | 62120 | Respiratory burst oxidase homolog protein C [Solanum tuberosum ] | 51.14 | 100.82 | 49.71 | 72.77 | 143.66 | 138.29 |
|  | 26933 | Respiratory burst oxidase homolog protein C [Solanum tuberosum ] | 25.01 | 42.11 | 18.85 | 36.14 | 62.56 | 55.04 |
|  | 24934 | Respiratory burst oxidase homolog protein C [Solanum tuberosum ] | 37.61 | 66.96 | 30.06 | 55.36 | 101.33 | 93.51 |
| RBOHD | 25003 | Respiratory burst oxidase homolog protein D [Solanum tuberosum ] | 157.42 | 250.29 | 171.31 | 241.25 | 379.27 | 386.99 |
| superoxide dismutase | 67103 | Extracellular superoxide dismutase [Cu-Zn] 2 [Dictyostelium discoideum] | 0.56 | 1.04 | 0.97 | 7.32 | 5.20 | 26.66 |
| Alcohol dehydrogenase (ADH) | 87628 | Probable cinnamyl alcohol dehydrogenase 1 [Oryza sativa subsp. Japonica] | 26.46 | 28.42 | 61.74 | 8.87 | 20.03 | 14.04 |
|  | 30916 | Probable cinnamyl alcohol dehydrogenase 1 [Oryza sativa subsp. Japonica] | 104.36 | 114.72 | 309.83 | 36.90 | 90.17 | 63.04 |
| **Pathogenesis-related protein** | 64043 | Pathogenesis-related protein PR-1 type [Sambucus nigra ] | 257.20 | 386.69 | 489.06 | 47.47 | 1111.83 | 438.44 |
|  | 62517 | Pathogenesis-related protein 1A [Nicotiana tabacum ] | - | 56.79 | - | - | - | - |
|  | 25523 | Pathogenesis-related protein 1C [Nicotiana tabacum] | 765.32 | 1981.00 | 1559.77 | 50.60 | 911.13 | 2125.84 |
|  | 62549 | Pathogenesis-related protein PR-4A [Nicotiana tabacum] | - | 124.91 | - | - | - | - |
|  | 63625 | Pathogenesis-related protein R major form [Nicotiana tabacum] | - | 106.17 | - | - | - | - |
|  | 26618 | Pathogenesis-related protein R minor form [Nicotiana tabacum] | 56.43 | 55.25 | 86.36 | 46.38 | 93.94 | 37.75 |
|  | 47039 | Pathogenesis-related protein STH-21 [Solanum tuberosum ] | - | 80.61 | - | - | - | - |
| beta-1,3-glucanase | 61724 | Lichenase [Nicotiana plumbaginifolia] | 0.35 | 538.30 | - | - | - | - |
|  | 40669 | Glucan endo-1,3-beta-glucosidase 14 [Arabidopsis thaliana] | 236.26 | 197.20 | 199.39 | 149.92 | 234.14 | 168.10 |
|  | 28689 | beta-1,3-glucanase [Elaeis guineensis] | 3.71 | 3.40 | 8.57 | 3.20 | 54.98 | 3.28 |
|  | 28688 | beta-1,3-glucanase [Elaeis guineensis] | 8.03 | 10.89 | 16.51 | 4.76 | 119.45 | 14.09 |
| Glucan endo-1,3-beta-glucosidase (glc) | 59112 | Glucan endo-1,3-beta-glucosidase GV [Hordeum vulgare] | - | 185.60 | - | - | - | - |
|  | 57964 | Glucan endo-1,3-beta-glucosidase GII [Hordeum vulgare ] | - | 370.16 | - | - | - | - |
|  | 54628 | Glucan endo-1,3-beta-glucosidase, basic vacuolar isoform [Hevea brasiliensis] | - | 569.40 | - | - | - | - |
|  | 36259 | Glucan endo-1,3-beta-glucosidase 8 [Arabidopsis thaliana] | 11.09 | 64.53 | 27.19 | 9.88 | 66.69 | 96.44 |
|  | 14820 | Glucan endo-1,3-beta-glucosidase [Triticum aestivum] | 26.97 | 100.87 | 52.42 | 5.75 | 98.39 | 141.17 |
| Exo-beta-1,3-glucanase | 19492 | Exo-beta-1,3-glucanase | 2.87 | 9.10 | 4.29 | - | 0.85 | 25.26 |
| chitinase | 25167 | chitinase [EC:3.2.1.14] | 15.71 | 203.08 | 299.04 | 9.94 | 175.42 | 13.11 |
|  | 69710 | chitinase [EC:3.2.1.14] | 8.81 | 171.10 | 204.64 | 9.64 | 129.82 | 8.78 |
| Thaumatin-Like Protein (TLP) | 5940 | Thaumatin-like protein [Arabidopsis thaliana ] | 35.95 | 162.93 | 52.42 | 11.51 | 142.51 | 150.73 |
|  | 23782 | Thaumatin-like protein [Arabidopsis thaliana ] | 19.17 | 128.77 | 37.42 | 4.93 | 97.87 | 60.56 |
| Metacaspase-4 | 743 | Metacaspase-4 [Arabidopsis thaliana ] | 30.90 | 17.03 | 31.21 | 35.16 | 16.65 | 15.71 |
|  | 37288 | Metacaspase-4 [Arabidopsis thaliana ] | 42.97 | 25.81 | 47.97 | 55.11 | 23.13 | 27.95 |
| Metacaspase-1 | 67249 | Metacaspase-1 [Yarrowia lipolytica ] | 0.50 | 0.56 | 0.37 | 0.24 | - | 8.50 |
|  | 64398 | Metacaspase-1 [Yarrowia lipolytica ] | - | - | - | - | 9.34 | 7.47 |
| Defender against apoptotic cell death (DAD1) | 28339 | Defender against cell death 1 [Solanum lycopersicum ] | 61.89 | 51.66 | 87.34 | 90.33 | 65.53 | 30.86 |
| BCL-2-associated athanogenes (BAGs) | 34122 | BCL-2 binding anthanogene-1 [Hordeum vulgare subsp. vulgare] | 15.22 | 7.36 | 15.95 | 18.12 | 9.50 | 4.68 |
|  | 23695 | ARABIDOPSIS THALIANA BCL-2-ASSOCIATED ATHANOGENE 1[Arabidopsis thaliana] | PSIS THALIANA BCL-2-ASS | CIATED ATHAN | GENE 1 | 29.06 | 12.39 | 12.41 |
|  | 14998 | BCL-2-associated athanogene 6 [Arabidopsis lyrata subsp. lyrata] | 22.08 | 7.64 | 13.92 | 89.43 | 25.86 | 4.83 |
|  | 14983 | BCL-2-associated athanogene 6 [Arabidopsis lyrata subsp. lyrata] | 39.67 | 6.58 | 7.88 | 129.73 | 37.89 | 8.67 |
| Apoptosis Inducing Factor (AIF) | 5852 | Apoptosis-inducing factor 2 [Xenopus tropicalis ] | 108.08 | 67.81 | 114.20 | 79.15 | 71.44 | 104.75 |
|  | 1565 | Apoptosis-inducing factor 2 [Xenopus tropicalis ] | 350.79 | 198.98 | 447.31 | 42.53 | 137.09 | 133.86 |
| Dynamin-related proteins (DRP) | 5826 | Dynamin-related protein 12A [Glycine max PE] | 80.80 | 66.07 | 58.97 | 56.94 | 44.76 | 39.03 |
|  | 16640 | Dynamin-related protein 1A [Arabidopsis thaliana ] | 118.52 | 97.09 | 84.69 | 96.73 | 69.37 | 65.44 |
|  | 5464 | Dynamin-related protein 1E [Arabidopsis thaliana ] | 73.84 | 40.21 | 44.82 | 124.65 | 50.63 | 34.91 |
|  | 7690 | Dynamin-related protein 3A [Arabidopsis thaliana ] | 44.10 | 27.46 | 26.54 | 44.71 | 27.33 | 13.21 |
| nitric oxide synthase (NOS) | 5744 | Nitric oxide synthase-interacting protein [Xenopus laevis ] | 89.94 | 76.32 | 81.13 | 103.43 | 80.21 | 89.06 |
| arginine decarboxylase (ADC) | 4618 | Arginine decarboxylase 1 [Oryza sativa subsp. japonica ] | 22.74 | 43.71 | 42.21 | 10.91 | 30.11 | 76.23 |
|  | 2443 | Arginine decarboxylase 1 [Oryza sativa subsp. japonica ] | 22.33 | 56.07 | 46.06 | 6.95 | 40.17 | 81.79 |
|  | 71175 | Arginine decarboxylase [Glycine max] | 138.14 | 152.85 | 165.27 | 109.82 | 93.80 | 229.16 |
|  | 35355 | Arginine decarboxylase [Glycine max] | 18.28 | 59.04 | 49.09 | 4.95 | 40.85 | 100.34 |
| Non-expressor of PR gene 1 (NPR1) | 36888 | Regulatory protein NPR1 [Arabidopsis thaliana ] | 112.01 | 121.34 | 97.42 | 106.75 | 131.50 | 147.87 |
| Pathogen-inducible salicylic acid glucosyltransferase (SAG) | 39802 | pathogen-inducible salicylic acid glucosyltransferase [EC:2.4.1.-] | 48.41 | 86.37 | 54.30 | 52.90 | 70.02 | 105.06 |
|  | 26044 | pathogen-inducible salicylic acid glucosyltransferase [EC:2.4.1.-] | 59.63 | 88.31 | 71.75 | 52.88 | 72.01 | 103.55 |
| phenylalanine ammonia lyase (PAL) | 43793 | Phenylalanine ammonia-lyase [Persea americana ] | 226.60 | 260.70 | 254.35 | 67.26 | 160.60 | 224.80 |
|  | 3356 | Phenylalanine ammonia-lyase 1 [Petroselinum crispum ] | 507.47 | 513.08 | 602.82 | 139.98 | 362.65 | 381.01 |
|  | 29644 | Phenylalanine ammonia-lyase 1 [Petroselinum crispum ] | 119.35 | 178.42 | 212.56 | 25.73 | 87.25 | 73.84 |
|  | 29643 | Phenylalanine ammonia-lyase [Bromheadia finlaysoniana ] | 337.51 | 312.21 | 381.20 | 122.43 | 215.69 | 190.31 |
|  | 29227 | Phenylalanine ammonia-lyase 3 [Petroselinum crispum ] | 161.48 | 123.53 | 185.30 | 60.66 | 98.79 | 76.42 |
|  | 27598 | Phenylalanine ammonia-lyase [Stylosanthes humilis ] | 119.19 | 161.74 | 187.27 | 15.03 | 71.42 | 71.51 |
|  | 19588 | Phenylalanine ammonia-lyase [Citrus limon ] | 305.74 | 321.60 | 327.78 | 72.51 | 204.41 | 163.22 |
| isochorismate synthase (ICS) | 35584 | isochorismate synthase 1 [Zea mays] | 229.78 | 135.17 | 342.99 | 186.08 | 120.38 | 104.80 |
|  | 18989 | isochorismate synthase 1 [Zea mays] | 65.73 | 34.52 | 61.20 | 53.64 | 37.63 | 36.97 |
| Lipoxygenase (LOX) | 8420 | Probable lipoxygenase 8, chloroplastic [Oryza sativa subsp. japonica ] | 4.92 | 93.95 | 25.54 | 0.29 | 114.78 | 134.38 |
|  | 21877 | Probable lipoxygenase 8, chloroplastic [Oryza sativa subsp. japonica ] | 3.43 | 112.01 | 36.68 | 0.30 | 138.96 | 176.51 |
|  | 12998 | Probable lipoxygenase 8, chloroplastic [Oryza sativa subsp. japonica ] | 2.02 | 32.39 | 6.06 | - | 38.11 | 64.80 |
|  | 79334 | Probable lipoxygenase 4 [Oryza sativa subsp. japonica ] | 133.50 | 66.87 | 112.95 | 93.34 | 64.92 | 27.58 |
|  | 20709 | Probable lipoxygenase 4 [Oryza sativa subsp. japonica ] | 1071.31 | 273.05 | 287.15 | 1066.16 | 310.12 | 177.26 |
|  | 32568 | Lipoxygenase B [Solanum lycopersicum ] | 163.62 | 311.24 | 196.24 | 81.77 | 231.73 | 257.11 |
|  | 30296 | Lipoxygenase B [Solanum lycopersicum ] | 80.34 | 144.88 | 59.83 | 60.60 | 109.68 | 133.32 |
|  | 3950 | Lipoxygenase A [Solanum lycopersicum ] | 307.95 | 593.97 | 311.89 | 174.95 | 486.29 | 540.88 |
|  | 29621 | Lipoxygenase A [Solanum lycopersicum ] | 385.64 | 110.45 | 114.63 | 434.69 | 126.95 | 77.33 |
|  | 70848 | Lipoxygenase 1 [Oryza sativa subsp. japonica ] | 247.87 | 474.12 | 282.71 | 139.20 | 401.13 | 479.83 |
|  | 37075 | Lipoxygenase 1 [Hordeum vulgare ] | 307.85 | 71.11 | 68.17 | 354.22 | 78.17 | 42.45 |
| Allene oxide synthase (AOS) | 58999 | Allene oxide synthase 2 [Oryza sativa subsp. japonica ] | 87.67 | 165.41 | 123.43 | 24.33 | 156.39 | 253.13 |
|  | 31788 | Allene oxide synthase 2 [Oryza sativa subsp. japonica ] | 90.38 | 178.98 | 145.20 | 31.82 | 132.36 | 319.76 |
|  | 18624 | Allene oxide synthase 2 [Oryza sativa subsp. japonica ] | 110.63 | 245.36 | 180.86 | 61.18 | 190.07 | 450.71 |
| ETHYLENE INSENSITIVE 3-like 1 | 7575 | ETHYLENE INSENSITIVE 3-like 1 protein [Arabidopsis thaliana ] | 112.03 | 320.45 | 143.00 | 176.14 | 421.67 | 393.89 |
| ethylene insensitive-like protein 3 | 15820 | Protein ETHYLENE INSENSITIVE 3 [Arabidopsis thaliana] | 178.26 | 493.87 | 209.65 | 294.09 | 706.48 | 505.92 |
| TIFY10B | 32908 | Protein TIFY 10B [Arabidopsis thaliana ] | 52.04 | 59.87 | 55.77 | 26.33 | 122.18 | 56.92 |
| 1-aminocyclopropane-1-carboxylic acid oxidase (ACO) | 69592 | 1-aminocyclopropane-1-carboxylate oxidase [Musa acuminata ] | 105.68 | 250.18 | 112.45 | 1.93 | 47.14 | 54.88 |
| 3-deoxy--D-arabino-heptulosonate 7-phosphate synthase (DAHPS) | 86551 | Phospho-2-dehydro-3-deoxyheptonate aldolase 2, chloroplastic [Oryza sativa subsp. japonica ] | 618.07 | 476.69 | 644.27 | 107.69 | 254.57 | 218.02 |
|  | 72822 | Phospho-2-dehydro-3-deoxyheptonate aldolase 1, chloroplastic [Oryza sativa subsp. japonica ] | 682.72 | 561.62 | 775.69 | 115.95 | 249.24 | 239.11 |
|  | 25826 | Phospho-2-dehydro-3-deoxyheptonate aldolase 1, chloroplastic [Oryza sativa subsp. japonica ] | 376.26 | 298.78 | 446.80 | 68.02 | 100.38 | 97.41 |
| 4-coumarate--CoA ligase (4CL) | 42290 | 4-coumarate--CoA ligase [Vanilla planifolia ] | 98.86 | 81.11 | 101.43 | 84.63 | 75.58 | 30.01 |
|  | 33315 | 4-coumarate--CoA ligase [Vanilla planifolia ] | 105.23 | 69.45 | 81.91 | 54.12 | 51.49 | 43.55 |
| polyphenol oxidase (PPO) | 86682 | Polyphenol oxidase, chloroplastic [Malus domestica] | 364.15 | 416.18 | 594.43 | 58.77 | 375.58 | 240.51 |
|  | 75541 | Polyphenol oxidase, chloroplastic [Vitis vinifera ] | 663.14 | 309.66 | 141.92 | 232.05 | 355.70 | 40.12 |
|  | 39128 | Polyphenol oxidase, chloroplastic [Malus domestica] | 202.72 | 192.94 | 338.32 | 41.41 | 145.57 | 125.65 |
| extension | 47818 | Extensin [Daucus carota] | 117.34 | 0.28 | - | - | - | - |
|  | 65932 | Extensin [Nicotiana tabacum] | - | - | - | 0.47 | 15.76 | 71.15 |
|  | 87888 | Retrotransposon-like protein 1 [Mus musculus] | 3.76 | 18.34 | 7.86 | 3.92 | 17.78 | 19.48 |
|  | 76011 | Extensin [Nicotiana tabacum] | - | - | - | - | 65.05 | 228.18 |
| glutathione-S-transferase(GST) | 28052 | Probable glutathione S-transferase BZ2 [Zea mays] | 243.56 | 127.55 | 328.39 | 91.87 | 132.90 | 132.07 |
|  | 74182 | Probable glutathione S-transferase GSTU6 [Oryza sativa subsp. Japonica] | 138.61 | 119.26 | 185.37 | 82.83 | 93.33 | 119.72 |
|  | 15746 | Probable glutathione S-transferase GSTU6 [Oryza sativa subsp. Japonica] | 168.92 | 301.53 | 397.89 | 61.34 | 258.01 | 334.19 |
|  | 23280 | Probable glutathione S-transferase parA [Nicotiana tabacum] | 107.27 | 49.27 | 221.05 | 15.08 | 43.43 | 42.46 |
|  | 2000 | Probable glutathione S-transferase parA [Nicotiana tabacum] | 71.79 | 40.56 | 120.25 | 28.15 | 44.22 | 35.29 |
|  | 17208 | Probable glutathione S-transferase parA [Nicotiana tabacum] | 104.74 | 82.30 | 177.83 | 39.16 | 95.23 | 62.90 |
| Caffeic acid 3-O-methyltransferase (COMT) | 8066 | Caffeic acid 3-O-methyltransferase [Zea mays] | 87.08 | 17.18 | 41.83 | 118.65 | 14.45 | 13.68 |
|  | 6196 | Caffeic acid 3-O-methyltransferase [Catharanthus roseus] | 141.44 | 49.37 | 170.48 | 7.01 | 122.01 | 13.11 |
|  | 41838 | Caffeic acid 3-O-methyltransferase [Saccharum officinarum] | 171.84 | 22.46 | 85.88 | 148.15 | 44.17 | 16.21 |
|  | 38767 | Caffeic acid 3-O-methyltransferase [Zea mays] | 122.29 | 47.62 | 126.49 | 6.71 | 100.36 | 4.24 |
|  | 36643 | Caffeic acid 3-O-methyltransferase [Eucalyptus gunnii] | 398.94 | 89.00 | 294.27 | 381.20 | 116.49 | 57.85 |
|  | 3521 | Caffeic acid 3-O-methyltransferase [Eucalyptus gunnii] | 126.18 | 93.23 | 219.09 | 1.35 | 25.21 | 12.71 |
|  | 26215 | Caffeic acid 3-O-methyltransferase [Zinnia elegans] | 259.30 | 54.72 | 76.68 | 228.43 | 60.40 | 31.32 |
|  | 1997 | Caffeic acid 3-O-methyltransferase [Zea mays ] | 132.39 | 120.85 | 242.42 | 3.58 | 33.71 | 17.35 |
|  | 15738 | Caffeic acid 3-O-methyltransferase [Catharanthus roseus] | 264.65 | 66.64 | 103.36 | 273.46 | 67.05 | 54.08 |
|  | 8806 | GATA transcription factor 5 [Arabidopsis thaliana ] | 6.09 | 8.28 | 5.28 | 21.76 | 9.51 | 5.72 |
| HSP90-like gene | 19459 | Heat shock protein 90 [Theileria annulata ] | 58.42 | 15.21 | 21.14 | 75.64 | 22.17 | 5.15 |
| nitrate reductase | 372 | Nitrate reductase [NADH] (Fragment) [Zea mays] | 37.65 | 42.69 | 134.34 | 1.03 | 22.87 | 10.09 |
| UDP-glucuronic acid decarboxylase 1 | 30873 | UDP-glucuronic acid decarboxylase 1 [Rattus norvegicus ] | 184.69 | 147.48 | 183.54 | 73.08 | 126.71 | 52.50 |
| UDP-glucuronic acid decarboxylase 1 | 17493 | UDP-glucuronic acid decarboxylase 1 [Rattus norvegicus ] | 292.27 | 242.38 | 304.17 | 86.47 | 211.87 | 84.93 |
| cellulose synthase | 318 | Probable cellulose synthase A catalytic subunit 1 [UDP-forming] [Oryza sativa subsp. Japonica] | 444.12 | 152.65 | 219.33 | 248.70 | 122.34 | 70.01 |
| cellulose synthase | 43394 | Probable cellulose synthase A catalytic subunit 2 [UDP-forming] [Oryza sativa subsp. Japonica] | 287.06 | 107.27 | 110.14 | 145.87 | 74.18 | 44.83 |
